# Gut–Brain Axis and Bile Acid Signaling: Linking Microbial Metabolism to Brain Function and Metabolic Regulation

**DOI:** 10.3390/ijms262412167

**Published:** 2025-12-18

**Authors:** Bojan Stanimirov, Maja Đanić, Nebojša Pavlović, Dragana Zaklan, Slavica Lazarević, Momir Mikov, Karmen Stankov

**Affiliations:** 1 Department of Biochemistry, Faculty of Medicine, University of Novi Sad, 21000 Novi Sad, Serbia; bojan.stanimirov@mf.uns.ac.rs (B.S.); karmen.stankov@mf.uns.ac.rs (K.S.); 2 Department of Pharmacology, Toxicology and Clinical Pharmacology, Faculty of Medicine, University of Novi Sad, 21000 Novi Sad, Serbia; maja.djanic@mf.uns.ac.rs (M.Đ.); slavica.lazarevic@mf.uns.ac.rs (S.L.); mikovmomir@gmail.com (M.M.); 3 Department of Pharmacy, Faculty of Medicine, University of Novi Sad, 21000 Novi Sad, Serbia; dragana.zaklan@mf.uns.ac.rs

**Keywords:** bile acids, gut–brain axis, gut microbiota, FXR, TGR5, metabolic regulation, neuroendocrine signaling

## Abstract

The gut–brain axis is a bidirectional communication network in which gut microbiota and their metabolites influence central nervous system (CNS) function. Among these metabolites, bile acids have emerged as key signaling molecules that modulate metabolic and neuroendocrine pathways. Microbiota-mediated modifications of bile acid composition affect receptors such as farnesoid X receptor (FXR)and Takeda G protein-coupled receptor 5 (TGR5), thereby influencing neuronal activity, appetite control, glucose metabolism, and energy balance. Emerging evidence indicates that bile acids act both directly on the CNS and indirectly via endocrine and immune mediators, linking microbial metabolism to brain function. By integrating microbiological, metabolic, and neuroendocrine perspectives, bile acids can be viewed as critical messengers in the communication between the gut microbiota and the CNS. The purpose of this review is thus to synthesize current mechanisms underlying these interactions and highlight their therapeutic potential in metabolic and neurodegenerative disorders.

## 1. Introduction

The regulation of energy homeostasis, appetite, and metabolic function is governed by a highly integrated network of communication between the gastrointestinal tract and the central nervous system (CNS), commonly referred to as the gut–brain axis. This axis comprises a complex interplay of neuronal, endocrine, immune, and metabolic pathways that enable the gut to sense and respond to both internal and external stimuli. Signals originating from the gut, including microbial metabolites, enteroendocrine hormones, and bile acids, convey information to the brain to influence feeding behavior, glucose metabolism, and systemic energy expenditure. Conversely, the CNS modulates gastrointestinal motility, secretion, and barrier function through autonomic and neuroendocrine pathways, thereby maintaining bidirectional communication essential for metabolic balance [1,2].

The intestinal microbiota has emerged as a key regulator within this network, shaping host physiology through the production of bioactive metabolites such as short-chain fatty acids, tryptophan derivatives, and secondary bile acids. Alterations in gut microbial composition have been associated with numerous metabolic and neuropsychiatric disorders, including obesity, type 2 diabetes, depression, and Parkinson’s disease but also with alterations in drug response [3,4,5,6,7]. Among the various molecular mediators of gut–brain communication, bile acids occupy a central position due to their dual role as digestive surfactants and potent signaling molecules [6]. Acting through receptors such as the farnesoid X receptor (FXR) and the G-protein-coupled bile acid receptor 1 (GPBAR1, also known as TGR5), bile acids influence not only hepatic and intestinal metabolism but also peripheral and central processes related to energy expenditure, thermogenesis, and neuroinflammation [8,9,10].

Emerging evidence suggests that bile acid signaling constitutes a crucial interface linking gut microbial activity to CNS function. Microbiota-driven transformations of bile acids modulate receptor activation and downstream signaling cascades that can impact neuronal activity, hypothalamic regulation of appetite, and even cognitive processes [11,12]. Understanding the dynamic relationship between microbial bile acid metabolism and neural signaling provides a framework for novel therapeutic interventions targeting the gut–brain axis in metabolic and neurodegenerative diseases.

This review aims to provide an integrated overview of the mechanisms by which gut microbiota and bile acid signaling contribute to communication along the gut–brain axis. Special emphasis is placed on the molecular pathways linking bile acid receptors to neuroendocrine and metabolic regulation, highlighting recent advances and potential implications for therapeutic modulation of this complex system.

## 2. The Gut–Brain Signaling Axis

The gut–brain axis represents as a complex bidirectional communication network essential for maintaining metabolic homeostasis is coordinated by the central nervous system. Sensory information regarding nutrient composition, volume, and caloric load, detected by specialized mechanisms along the gastrointestinal tract, is transduced into neuronal, hormonal, and immune signals directed to multiple brain regions, including the brainstem and hypothalamus [13]. Higher-order integration within these centers orchestrates both acute and long-term adjustments in energy intake and expenditure, thereby maintaining systemic homeostasis [14].

The intestinal microbiota and its metabolites are integral to this axis, modulating feeding behavior and satiety primarily through endocrine and immune pathways, often mediated by the vagus nerve [15]. Microbial metabolites, including short-chain fatty acids (SCFAs), secondary bile acids, and tryptophan derivatives, transmit signals via interactions with enteroendocrine cells, enterochromaffin cells, and the mucosal immune system [2,16,17,18,19]. Some metabolites enter systemic circulation and cross the blood–brain barrier (BBB), whereas others activate central circuits via vagal or spinal afferent signaling [20]. Furthermore, gut microorganisms can synthesize or contribute to the synthesis of several neuroactive compounds, such as γ-aminobutyric acid (GABA), glutamate, 5-hydroxytryptamine (serotonin, 5-HT), norepinephrine, and dopamine. However, whether these molecules reach relevant neural receptors at physiologically effective concentrations remains uncertain [21,22,23,24].

## 3. Neural Pathways Between Gut and Brain

The proximal small intestine, the principal site of nutrient sensing, is richly innervated by vagal and splanchnic fibers, with afferent neurons greatly outnumbering efferents, underscoring the dominance of sensory signaling from the gut to the brain [25]. Vagal afferents detect luminal and mucosal chemical cues, respond to gut hormones and neurotransmitters, and relay information on nutrient availability and microbial metabolites to the central nervous system [26]. These afferents terminate within the lamina propria of intestinal villi, adjacent to the basolateral membranes of enteroendocrine cells, and express receptors for gut-derived peptides such as cholecystokinin (CCK), glucagon-like peptide 1 (GLP-1), and peptide YY_3–36_ (PYY). Activation of these receptors triggers neuronal excitation [27]. In parallel, nutrient-induced hormone secretion indirectly activates vagal and spinal afferents through enteric neurons situated near both enteroendocrine cells and afferent terminals [28,29,30]. Recent findings identifying synaptic connections between enteroendocrine cells and vagal neurons provide a mechanistic basis for direct sensing of luminal contents, including nutrients and microbial products [31]. Enteric neurons in both humans and rodents express Toll-like receptors (TLRs)—TLR3 and TLR7, which recognize viral RNA, and TLR2 and TLR4, which detect bacterial components such as peptidoglycan and lipopolysaccharide, highlighting a critical role for microbial antigens in maintaining enteric nervous system homeostasis [32,33,34]. Receptors for microbial metabolites, notably free fatty acid receptors 2 and 3 (FFAR2/GPR43 and FFAR3/GPR41), are expressed on enteroendocrine L-cells and respond to SCFAs by activating vagal afferents, thereby relaying metabolic information to the brain [15,35,36].

**Figure 1:**
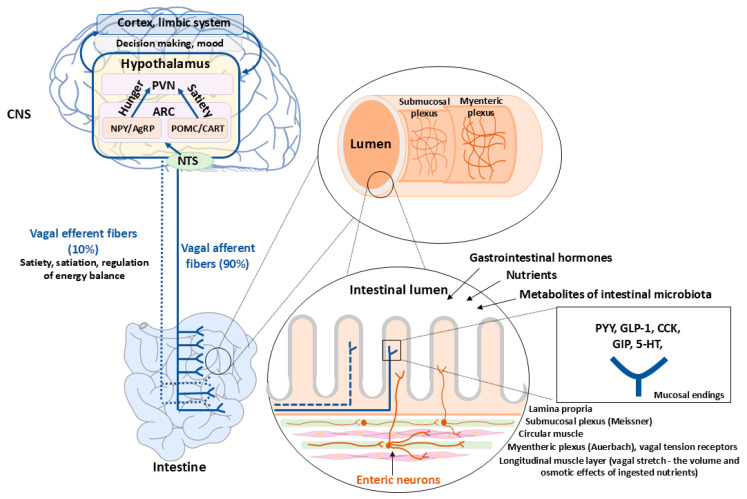
The Vagus Nerve and Enteric Nervous System as Bidirectional Mediators of Satiety and Metabolism. This figure illustrates the role of the vagus nerve and enteric nervous system in gut–brain communication. Sensory vagal afferents transmit signals derived from nutrients, gut hormones, enteroendocrine cells, immune mediators, microbial metabolites, and gut microbiota to the nucleus tractus solitarius (NTS), which integrates information and relays it to higher-order brain regions involved in mood, cognitive processing, and metabolic regulation. Abbreviations: AgRP—agouti-related peptide, ARC—arcuate nucleus, CART—cocaine- and amphetamine-regulated transcript, GIP—glucose-dependent insulinotropic polypeptide, NPY—neuropeptide Y, PVN—paraventricular nucleus, POMC—pro-opiomelanocortin.

Following food intake, nutrient-related sensory information is conveyed by gastrointestinal vagal and spinal afferents to the nucleus tractus solitarius (NTS). NTS neurons project to higher-order centers, including the hypothalamus—the principal integrative hub regulating orexigenic and anorexigenic responses and adaptive metabolic processes [37,38,39]. The arcuate, paraventricular, ventromedial, and dorsomedial nuclei, as well as the lateral hypothalamus, form interconnected circuits that coordinate feeding behavior and energy balance [40,41]. Within the arcuate nucleus, two functionally antagonistic neuronal populations: neuropeptide Y (NPY)/agouti-related peptide (AgRP) neurons and pro-opiomelanocortin (POMC)/cocaine- and amphetamine-regulated transcript (CART) neurons, mediate opposing effects on appetite and metabolism. NPY/AgRP neurons promote feeding and reduce energy expenditure, whereas POMC/CART neurons release α-melanocyte-stimulating hormone (α-MSH) and CART to suppress appetite and increase energy utilization (Figure 1) [42,43]. Energy homeostasis thus reflects the dynamic equilibrium between these neuronal populations. Peripheral signals, including gut-derived hormones, can directly influence hypothalamic circuits owing to the incomplete BBB in the arcuate nucleus and area postrema, which permits access of circulating peptides that rise postprandially [44].

## 4. Enteroendocrine Cell Signaling

**Figure 2:**
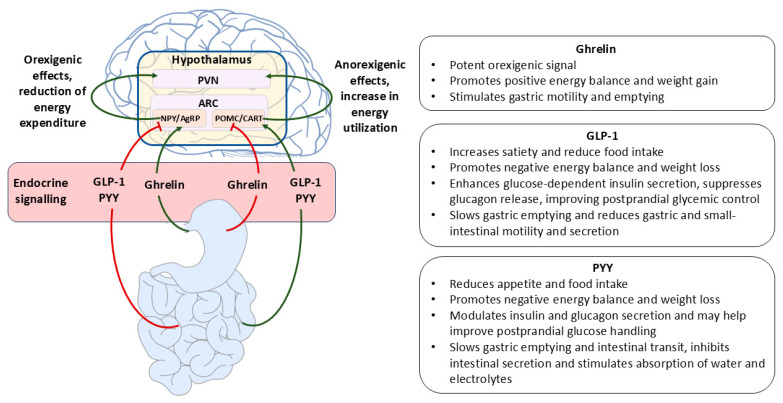
Gut Hormone Control of Appetite and Metabolism through Endocrine Gut–Brain Communication. Schematic representation of endocrine pathways through which gut hormones regulate energy balance and gastrointestinal function. Ghrelin, secreted from the stomach in the fasting state, enters the circulation and activates orexigenic hypothalamic signaling, thereby stimulating hunger, promoting energy intake, and favoring positive energy balance. In contrast, postprandial release of GLP-1 and PYY from the distal intestine acts via the bloodstream to enhance satiety, slow gastric emptying, and improve postprandial glucose regulation through metabolic effects in peripheral organs. The interplay between these opposing endocrine signals modulates central appetite control and gastrointestinal physiology. Persistent imbalance-characterized by insufficient satiety signaling or exaggerated hunger drive-contributes to obesity, insulin resistance, and gastrointestinal motility disturbances, highlighting these hormones as critical therapeutic targets along the gut–brain axis. Abbreviations: AgRP—agouti-related peptide, ARC—arcuate nucleus, CART—cocaine- and amphetamine-regulated transcript, GLP-1—glucagon-like peptide 1, NPY—neuropeptide Y, PVN—paraventricular nucleus, POMC—pro-opiomelanocortin.

The signaling pathway from the intestine to the central nervous system (CNS) is initiated by nutrient-induced hormone secretion from specialized chemosensory epithelial cells known as enteroendocrine cells. These polarized cells, interspersed among enterocytes and comprising approximately 1% of the intestinal epithelium, express receptors for pre-absorptive nutrients and a wide range of luminal and circulating factors, enabling them to secrete intestinal hormones in response to nutrient stimuli [45]. Digested luminal nutrients interact with microvilli on the apical surface of enteroendocrine cells to trigger hormone release at the basolateral membrane. Nutrient sensing occurs via G-protein-coupled receptors (GPRs) expressed on the apical membrane, whose activation induces the secretion of more than 20 biologically active peptides targeting various peripheral and central organs. Following secretion, these peptides act both as endocrine hormones, regulating distant organs such as the pancreas and brain, and as paracrine signals modulating nearby intestinal cells and neurons of the enteric nervous system (ENS) [46]. Increasing evidence suggests that gut peptides primarily signal to the brain through paracrine mechanisms acting on receptors expressed in afferent neurons that innervate the gut wall [39]. Enteroendocrine cells subtypes differ in both their anatomical distribution and the peptide hormones they secrete. The gastric mucosa contains ghrelin-producing X/A-like cells and leptin-secreting chief cells; the proximal small intestine harbors I cells and K cells, which release cholecystokinin (CCK) and glucose-dependent insulinotropic polypeptide (GIP), respectively; while the distal small intestine contains L cells producing glucagon-like peptides 1 and 2 (GLP-1/2), oxyntomodulin, and peptide YY (PYY). L cells are distributed throughout the gastrointestinal tract, with the highest density in the ileum and colon. Gut peptides released in response to chemical or mechanical stimulation can either enter the circulation, reaching central structures involved in appetite regulation, such as the nucleus tractus solitarius, or act locally by activating vagal afferents within the gut or hepatic portal area, thereby eliciting central satiety signals [47]. Circulating concentrations of PYY, GLP-1, and oxyntomodulin rise biphasically after food intake, with an early peak at approximately 15 min and a second peak around 90 min postprandially [48]. The early response likely reflects neural (vagal) and/or hormonal signaling, whereas the delayed peak results from direct nutrient contact with L cells in the ileum and colon [49]. Figure 2 illustrates the endocrine gut hormone pathways that integrate nutrient sensing with central regulation of appetite and metabolic balance, with relevance to obesity and gastrointestinal dysfunction.

GLP-1 is a neuropeptide primarily secreted by L cells in the ileum and colon in response to carbohydrate, lipid, and protein ingestion [50]. It is also synthesized in the CNS by a subset of preproglucagon-expressing neurons located in the nucleus tractus solitarius and the intermediate reticular nucleus of the hindbrain. Through post-translational processing of the preproglucagon gene, GLP-1 acts on its cognate receptor (GLP-1R), a G-protein-coupled receptor abundantly expressed in the CNS, gastrointestinal tract, and pancreas [51]. Systemic or central administration of GLP-1 activates satiety centers—particularly the arcuate and paraventricular nuclei, the nucleus tractus solitarius, and the area postrema, thereby suppressing hunger [52]. GLP-1 functions as a potent incretin hormone by stimulating insulin secretion and protecting pancreatic β-cells from apoptosis via receptor-mediated signaling [53]. In addition, GLP-1 slows gastric emptying, inhibits gastric acid secretion, reduces food intake, enhances satiety, and contributes to overall energy homeostasis [54].

GLP-1 is rapidly degraded and inactivated by the enzyme dipeptidyl peptidase-4 (DPP-4), with only about 10% of gut-derived GLP-1 reaching systemic circulation [55,56]. Following a meal, GLP-1 concentrations in lymph rise rapidly and remain elevated for prolonged periods, likely due to the lower expression of DPP-4 in lymph compared with plasma. Postprandial GLP-1 levels are highest within the intestinal lamina propria and hepatic portal vein [57,58]. Activation of vagal afferents in the lamina propria conveys GLP-1-mediated satiety signals to the CNS (59), whereas GLP-1 signaling via vagal afferents in the hepatic portal vein contributes to glucose homeostasis by modulating hepatoportal glucose sensors [59]. Together, these findings indicate that intestinal L cells form a neuroepithelial circuit that directly synapses with sensory afferents, facilitating the transmission of nutrient-derived sensory information from the intestinal lumen to the CNS.

## 5. Bile Acid Metabolism in the CNS

**Figure 3:**
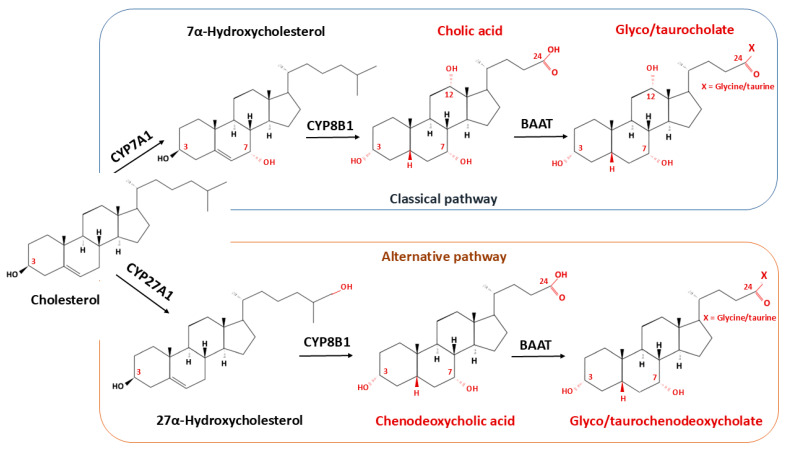
Hepatic Biosynthesis of Primary Bile Acids. Simplified overview of the two major biosynthetic pathways of primary bile acids in the liver. In the classic (neutral) pathway, cholesterol is converted to 7α-hydroxycholesterol by CYP7A1, followed by a series of mitochondrial and microsomal reactions leading to the formation of cholic acid (CA) and chenodeoxycholic acid (CDCA). In the alternative (acidic) pathway, CYP27A1 initiates mitochondrial side-chain oxidation, with subsequent 7α-hydroxylation by CYP7B1. Newly synthesized bile acids are conjugated with taurine or glycine, transported into bile canaliculi, and secreted into the intestine to participate in lipid digestion and enterohepatic cycling. Abbreviations: BAAT—Bile acid-CoA:amino acid N-acyltransferase, CYP7A1—Cholesterol 7α-hydroxylase, CYP8B1—Sterol 12α-hydroxylase, CYP27A1—Sterol 27-hydroxylase, CYP7B1—Oxysterol 7α-hydroxylase.

**Figure 4:**
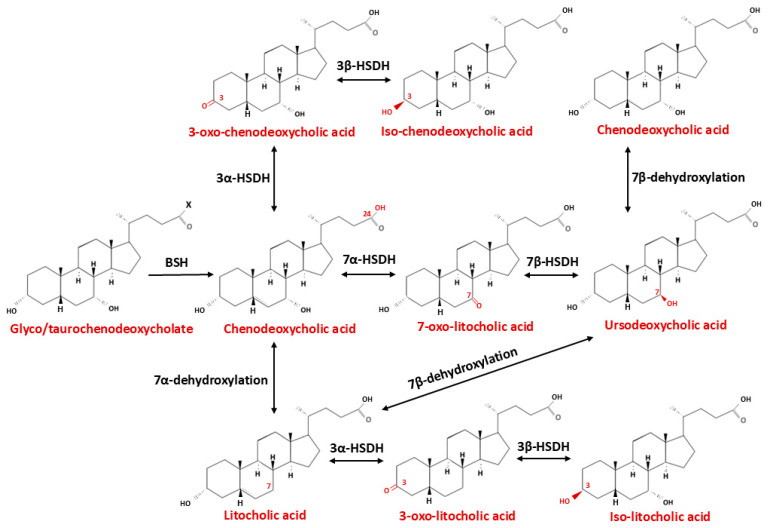
Microbiota-Driven Biotransformation of Chenodeoxycholic Acid in the Intestine. Schematic representation of the major microbial enzymatic transformations of chenodeoxycholic acid in the human intestine. Following secretion into the intestinal lumen, chenodeoxycholic acid undergoes deconjugation by bile salt hydrolases (BSH), followed by 7α-dehydroxylation and other microbiota-dependent reactions that convert primary bile acids into secondary bile acids. These biotransformation pathways shape the intestinal bile acid pool, modulate host–microbe interactions, and influence enterohepatic circulation and gut physiology. Abbreviations: HSDH—Hydroxysteroid dehydrogenase.

**Figure 5:**
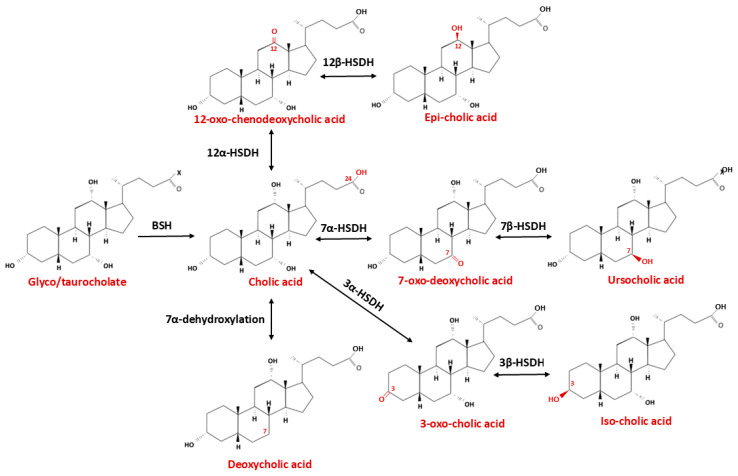
Microbiota-Driven Biotransformation of Cholic Acid in the Intestine. Abbreviations: BSH—Bile salt hydrolase, HSDH—Hydroxysteroid dehydrogenase.

The composition of bile acids in the brain, similar to that in the liver, is complex and encompasses multiple primary and secondary, conjugated and unconjugated species. The key enzymatic steps and regulatory pathways of hepatic bile acid biosynthesis are summarized in Figure 3. The brain contains bile acids derived either from systemic circulation or from local synthesis within the central nervous system (CNS) [60]. Secondary bile acids are generated in the intestine through microbial biotransformations including deconjugation, dihydroxylation, dehydrogenation, oxidation–reduction, desulfation, esterification, epimerization, and amidation of primary bile acids, and are not produced de novo within the CNS [61]. The reactions of biotransformation of primary bile acid CDCA and CA by intestinal microbiota are shown on Figure 4 and Figure 5, respectively. Although deconjugation reduces active intestinal reuptake, a small fraction (up to 10%) of deconjugated bile acids can passively diffuse across enterocytes into the enterohepatic circulation, enabling them to function as signaling molecules. In humans, systemic plasma bile acid concentrations fluctuate postprandially, following a diurnal rhythm ranging from approximately 5 μM to 15 μM depending on nutrient intake [62]. These transient oscillations suggest that circulating bile acids may act as postprandial metabolic signals conveying information on nutrient and energy availability.

### 5.1. Bile Acids and Blood–Brain Barrier

Circulating bile acids reach the brain via the internal carotid and vertebral arteries. The concentrations of cholic acid (CA), chenodeoxycholic acid (CDCA), and deoxycholic acid (DCA) in brain tissue correlate with their serum levels, consistent with the ability of unconjugated bile acids to passively diffuse through the phospholipid bilayers of the BBB [63,64,65,66]. Similarly, unconjugated ursodeoxycholic acid (UDCA) has been shown to cross the BBB in a dose-dependent manner following oral administration in patients with amyotrophic lateral sclerosis (ALS) [67]. In contrast, bile acids conjugated with glycine or taurine exhibit increased amphiphilicity and solubility, rendering them membrane-impermeable and dependent on active transport to enter the brain [68,69]. Transporters such as OSTα/β, OATP, ASBT, NTCP, and BSEP are expressed at the BBB and within the brain, supporting the concept that cerebral bile acids may originate from both hepatic and intestinal sources [70]. At pathologically high concentrations (around 1.5 mM), as observed in cholestasis, bile acids increase BBB permeability through detergent-like effects that disrupt cell membranes [71,72]. Even at lower physiological concentrations (0.2–1.5 mM), bile acids can subtly modulate BBB permeability [71]. Mechanistically, circulating bile acids may enhance BBB permeability by promoting Rac1-dependent phosphorylation of the tight-junction protein occludin, thereby weakening tight junction integrity and allowing bile acids and other solutes to diffuse into the brain [64]. Conversely, UDCA and its glycine conjugate, glycoursodeoxycholic acid (GUDCA), exert cytoprotective effects on brain capillary endothelial cells by reducing apoptosis [66].

Metabolomic analyses have confirmed the presence of both conjugated and unconjugated bile acids in the brains of humans and rodents [60,63,73]. For example, unconjugated CA, CDCA, and DCA have been identified in the cytoplasmic fraction of rat brain, with CDCA predominantly existing in a protein-bound form [74]. Another study reported CA as the most abundant bile acid species in rat brain, with lower levels of protein-bound CDCA [63].

Specific transporters such as the apical sodium-dependent bile acid transporter (ASBT), expressed in the hypothalamus and frontal cortex, mediate the uptake of peripheral bile acids into the CNS and facilitate neuronal bile acid transport [70]. ASBT activity has been shown to promote the influx of bile acids into hypothalamic neurons, modulating the hypothalamic–pituitary–adrenal (HPA) axis [65,75]. Unconjugated bile acids (CA, CDCA, and DCA) appear to enter the brain predominantly via passive diffusion, as their cerebral levels correlate with serum concentrations and isotopically labeled d4-CA and d4-CDCA administered intraperitoneally have been detected in brain tissue [63]. The extent of bile acid diffusion across the BBB is determined primarily by their degree of hydrophobicity.

### 5.2. Bile Acid Synthesis in the Brain

Bile acids can also be synthesized locally within the brain, predominantly in astrocytes and neurons, as all genes encoding cytochrome P450 enzymes required for the alternative (acidic) pathway of bile acid synthesis, CYP8B1, CYP27A1, and CYP7B1, are expressed in neural tissue [74,76,77]. This intracerebral biosynthetic route is thought to contribute to the clearance of excess cholesterol from the brain [78]. Neuron-specific sterol 24-hydroxylase (CYP46A1), which is highly expressed in the cerebral cortex, hippocampus, amygdala, caudate nucleus, putamen, and frontal lobe, catalyzes the conversion of cholesterol into the more soluble oxysterol 24(S)-hydroxycholesterol, which is subsequently metabolized to chenodeoxycholic acid (CDCA) [79]. The 24(S)-hydroxycholesterol molecule can cross the BBB via lipoprotein-mediated transport through the ATP-binding cassette transporter ABCA1, enter the systemic circulation, and reach the liver, where it is incorporated into the alternative bile acid synthesis pathway via oxysterol 7α-hydroxylase II (CYP39A1) [80].

The rate of CDCA synthesis through this alternative pathway is influenced by the availability of 24(S)-hydroxycholesterol, which serves as an indirect indicator of the cerebral cholesterol pool. This mechanism reflects a bidirectional crosstalk between central and peripheral bile acid metabolism. Under physiological conditions, bile acid concentrations in the brain closely mirror those in systemic circulation, suggesting that peripheral uptake plays a more dominant role in determining cerebral bile acid levels than local synthesis [81]. Secondary bile acid species detected in the brain are believed to originate primarily from the intestine, as the enzymes required for their production are expressed exclusively by the gut microbiota. Consequently, the ratio of primary to secondary bile acids in the CNS is largely determined by the composition and metabolic activity of the intestinal microbiome [82].

## 6. Direct and Indirect Bile Acid Signaling in the CNS

Bile acids present in the brain arise from two mechanistically distinct sources peripheral uptake across the BBB and de novo synthesis within neural tissue, and emerging evidence indicates that these origins confer divergent effects on CNS signaling. Peripherally derived bile acids primarily reflect hepatic metabolism and microbiota-dependent transformations, entering the CNS through passive diffusion (unconjugated species such as CA, CDCA, DCA) or active transport mediated by ASBT, OATP, and OSTα/β (conjugated species), thereby coupling central signaling to systemic metabolic states, circadian rhythms, and composition of intestinal microbiota [64,65,83]. These circulating bile acids tend to activate receptors such as TGR5 and S1PR2 in a concentration-dependent manner, often promoting microglial activation, neuroinflammation, or HPA axis suppression under cholestatic conditions [64,65].

In contrast, locally synthesized bile acids, derived from neuronal and glial expression of CYP27A1, CYP7B1, and CYP46A1, produce a more spatially restricted and tightly regulated intracerebral pool that appears to preferentially modulate cholesterol turnover, cytoprotective pathways, and autocrine/paracrine receptor signaling [73]. For example, 24(S)-hydroxycholesterol, produces by CYP46A1, is a precursor for CDCA synthesis within the brain and can selectively influence FXR signaling in hippocampal and cortical neurons, impacting synaptic plasticity, neurotransmission, and neuroprotection independently of peripheral bile acid fluctuations [76,84]. These differences imply that peripherally derived bile acids serve predominantly as systemic metabolic signals that inform the CNS about hepatic and intestinal status, while locally synthesized bile acids function as intrinsic neuromodulators involved in cholesterol homeostasis, neuronal excitability, and region-specific receptor activation. Recent human and animal studies indicate that the relative contribution of these pathways determines ligand identity, primary versus secondary, conjugated versus unconjugated, as well as transporter engagement (ASBT, OATP, OSTα/β) and regional distribution within CNS structures [82,85,86]. Importantly, these differences shape downstream receptor activation: peripherally derived hydrophobic or unconjugated bile acids more effectively engage membrane receptors such as TGR5 and S1PR2, producing rapid non-genomic signaling effects, whereas locally synthesized oxysterol-derived ligands preferentially modulate nuclear receptors including FXR and PXR, influencing transcriptional and metabolic programs within neurons and glia (82).

Human studies demonstrate that fluctuations in circulating bile acids modestly influence CNS-related endpoints—such as HPA axis tone, neuroinflammation and autonomic output, reflecting limited but physiologically relevant brain penetration [87,88]. Conversely, experimental upregulation of neuronal CYP46A1 in rodent models enhances the generation of 24-hydroxycholesterol, shifts intracerebral ligand profiles toward FXR-activating species and elicits neuroprotective transcriptional responses [86].

Understanding the relative contribution of these two sources is therefore essential for interpreting the mechanisms of bile acid–dependent neuromodulation discussed in the following sections.

### 6.1. Direct Bile Acid Signaling in the Brain and the Regulation of Energy Homeostasis

**Figure 6:**
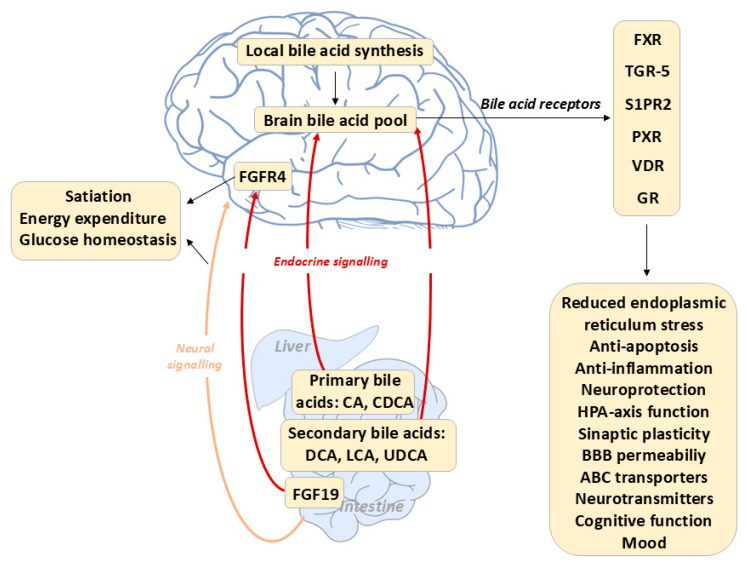
Emerging Neuromodulatory Roles of Bile Acids Along the Gut–Brain Axis. Schematic representation of the expanding role of bile acids in modulating central nervous system (CNS) function. Primary bile acids synthesized in the liver and secondary bile acid species modified through intestinal microbial metabolism enter the circulation and act as endocrine messengers that activate bile acid receptors expressed in metabolic tissues and neural structures. The cerebral bile acid pool comprises both locally synthesized bile acids within the CNS and peripherally derived bile acids that reach the brain via the systemic circulation. By activating nuclear and membrane-bound receptors in the CNS, bile acids regulate neuroinflammatory signaling, synaptic activity, mitochondrial function, and synaptic transmission. In addition to endocrine actions, bile acids can influence brain function indirectly through neural pathways, including vagal afferent signaling, as well as through bile acid dependent enterokine FGF19. Disturbances in bile acid homeostasis and receptor signaling are increasingly linked to impaired brain function, underscoring their emerging potential as therapeutic targets within the gut–brain axis. Abbreviations: ABC—ATP-binding cassette transport proteins, BBB—Blood–brain barrier, CA—Cholic Acid, CDCA—Chenodeoxycholic Acid, DCA—Deoxycholic Acid, FGF—Fibroblast Growth Factor, FXR—Farnesoid X Receptor, GR—Glucocorticoid receptor, PXR—Pregnane X receptor, S1PR2—Sphingosine-1-phosphate receptor 2, TGR5—Takeda G-Protein Coupled Receptor-5, UDCA—Ursodeoxycholic acid, VDR—Vitamin D receptor.

Although bile acid–dependent signaling has been extensively characterized in hepatic and intestinal tissues, its roles within the central nervous system (CNS) have only recently begun to emerge. A growing body of evidence now supports the concept that bile acids exert neuroactive effects and directly influence brain function. Figure 6 summarizes the newly identified neurophysiological actions of bile acids and their therapeutic relevance in disorders of the gut–brain axis.

The signaling activity of bile acids arises from their capacity to bind to and modulate several nuclear and membrane receptors, including the nuclear FXR, pregnane X receptor (PXR), vitamin D receptor (VDR), and glucocorticoid receptor (GR), as well as the membrane-bound G protein-coupled receptors Takeda G protein-coupled receptor 5 (TGR5, also known as GPBAR1) and sphingosine 1-phosphate receptor 2 (S1PR2) (Table 1) [72,89]. While these receptors are abundantly expressed in the liver and intestine, they are also detected across multiple tissues, including the brain [90]. The affinities of primary, secondary, and conjugated bile acids for these receptors differ markedly. Although bile acid receptors are expressed in neural tissue, the extent to which they mediate direct neurophysiological effects remains incompletely understood. Functional FXR expression has been confirmed in human and murine brain tissue, primarily localized to cortical neurons, though its specific neuronal functions remain to be fully defined [84]. In murine models, FXR gene expression is significantly upregulated in the hippocampus following stress-induced depression, whereas genetic disruption of FXR signaling ameliorates depression-like behavior [91]. Studies in FXR-null mice further indicate that loss of FXR disrupts glutamatergic, GABAergic, serotonergic, and noradrenergic neurotransmission in the hippocampus and cerebellum, resulting in impaired cognitive performance and motor coordination [92]. Normally expressed at low levels in cortical neurons, FXR is upregulated under neuroinflammatory conditions [93]. Collectively, these findings suggest that FXR contributes to neurotransmitter homeostasis and behavioral regulation, likely by modulating systemic bile acid concentrations that secondarily influence cerebral bile acid levels [92].

Intracerebroventricular administration of tauroLCA acid increases fat oxidation, reduces adiposity, and promotes browning of subcutaneous white adipose tissue, accompanied by increased fatty acid uptake in brown adipose tissue. By contrast, central administration of the selective FXR agonist GW4064 exerts no measurable effects on energy metabolism, body composition, or bile acid concentrations, indicating an FXR-independent mechanism [94]. Conversely, direct hypothalamic activation of FXR by GW4064 decreases sympathetic nervous system activity and energy expenditure, effects that are abolished in FXR-deficient mice, confirming receptor-specific actions [95]. Since taurolithocholate is a selective TGR5 agonist with no FXR activity, these findings suggest that FXR and TGR5 exert opposing influences on central energy regulation, with TGR5 promoting and FXR reducing energy expenditure. Thus, therapeutic modulation of hypothalamic TGR5 activation or FXR inhibition may represent strategies for preventing metabolic disorders. Low-level TGR5 mRNA expression has been detected in the hippocampus, cerebellum, and hypothalamus of rabbits [68], and further studies confirmed TGR5 mRNA and protein expression in the mouse brain, particularly in the hippocampus and frontal cortex, as well as immunofluorescent localization in neurons and astrocytes across rat and human neural tissue [96,97]. However, comprehensive mapping of TGR5 distribution in the human brain remains lacking. Within the CNS, TGR5 signaling attenuates neuroinflammation and microglial activation [98].

**Figure 7:**
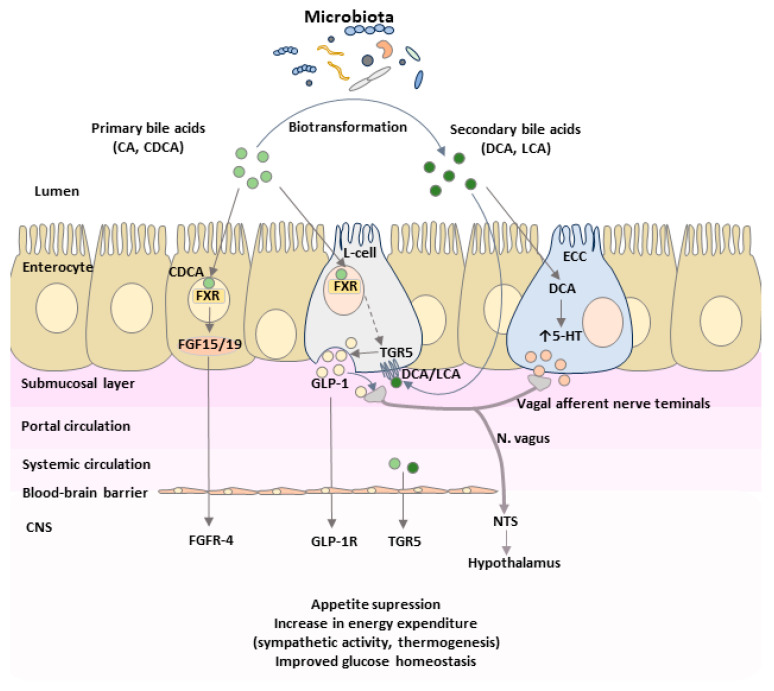
Bile acids and the microbiota–gut–brain signaling axis in the regulation of metabolic and energy homeostasis. Primary and secondary bile acids can signal from intestine to the CNS via the direct or indirect pathways. Following reabsorption bile acids reach the systemic circulation, and cross the blood–brain barrier to interact with receptors in the brain. Bile acids activate TGR5 in the nucleus arcuatus of the hypothalamus exerting anorexigenic effect. Within the enterocytes bile acids activate the nuclear receptor FXR to promote FGF15/19 production. FGF15/19 enters the systemic circulation and crosses the blood–brain barrier to interact with FGF receptors in the brain. The central actions of FGF15/19 are associated with glucose metabolism and energy homeostasis. Activation of TGR5 by bile acids in enteroendocrine L cells triggers the production of GLP-1. GLP-1 might interact with GLP-1 receptors located on afferent terminals of the vagus nerve in the lamina propria, exerting the inhibitory effect on food intake and energy homeostasis via the vagal-brainstem-hypothalamic pathway. Abbreviations: CA—Cholic Acid, CDCA—Chenodeoxycholic Acid, DCA—Deoxycholic Acid, ECC—Enterochromaffin Cell, FGF—Fibroblast Growth Factor, FXR—Farnesoid X Receptor, GLP-1—Glucagon-Like Peptide-1, 5-HT—5-Hydroxytryptamine, LCA—Litocholic Acid, NTS—Nucleus Tractus Solitarius, TGR5—Takeda G-Protein Coupled Receptor-5.

Postprandially, bile acids can access the brain and engage a TGR5-dependent negative feedback loop that modulates satiety in response to nutrient intake (Figure 7) [82]. Both peripheral and central administration of a bile acid mixture (sodium salts of tauroCA, glycoCA, deoxycholic, and CA) or the TGR5-specific agonist INT-777 induce anorexigenic effects by suppressing orexigenic neuropeptide expression in agouti-related peptide/neuropeptide Y (AGRP/NPY) hypothalamic neurons. In contrast, neuron-specific or AGRP-neuron TGR5 deletion markedly increases food intake [99]. Central administration of bile acids or the TGR5 agonist CCDC (3-(2-Chlorophenyl)-N-(4-chlorophenyl)-N,5-dimethyl-4-isoxazolecarboxamide) in obese mice enhances sympathetic tone, promotes negative energy balance, and reduces body weight and adiposity [100]. Conversely, downregulation of hypothalamic TGR5 expression enhances obesity susceptibility, indicating that TGR5 signaling in the mediobasal hypothalamus is essential for protecting against diet-induced obesity. Activation of TGR5 also suppresses appetite by stimulating neurons in the nodose ganglia through the hypothalamic POMC/CART pathway [101], whereas FXR activation (via GW4064) does not elicit this effect.

While FXR activation is critical for maintaining cognitive and motor integrity, and TGR5 activation exerts anti-inflammatory and anti-obesity effects, signaling through S1PR2 appears to have deleterious outcomes. S1PR2, expressed in the hippocampus, cerebellum, and motor cortex [102] is activated by the conjugated bile acid tauroCA, which in murine models of hepatic encephalopathy induces neuroinflammatory gene expression. Pharmacological inhibition of S1PR2 attenuates inflammation, preserves BBB integrity, and prevents leukocyte infiltration [103,104]. Thus, targeting S1PR2 signaling or reducing bile acid accumulation may offer therapeutic benefit in hepatic encephalopathy and other neuroinflammatory disorders.

In addition, PXR mRNA and protein have been detected in porcine brain endothelial cells and primary hippocampal neurons [105], while VDR is expressed in both neurons and astrocytes, with highest expression levels in the hypothalamus and substantia nigra [106], However, the physiological relevance of bile acid signaling through these receptors in the CNS remains poorly defined [65]. Bile acids can also act as weak activators of the glucocorticoid receptor (GR) in the brain. Under conditions of extrahepatic biliary obstruction or cholestasis, tauroCA and glycoCDCA cross the BBB, facilitated by increased tight junction permeability [64]. Once in the hypothalamus, bile acids are taken up by ASBT-expressing neurons, where they suppress the HPA axis by activating GR and reducing corticotropin-releasing hormone (CRH) expression [75]. This suppression is prevented by dietary bile acid sequestration with cholestyramine [65]. Similarly, central administration of FGF19 decreases circulating adrenocorticotropic hormone and corticosterone levels, further implicating bile acid signaling in HPA axis modulation [107]. Together, impaired hepatic glucocorticoid clearance and direct central bile acid and FGF19 actions contribute to HPA axis dysregulation during cholestasis.

Finally, bile acids may modulate neuronal excitability, proliferation, and differentiation through interactions with neurotransmitter receptors such as N-methyl-D-aspartate (NMDA) and γ-aminobutyric acid type A (GABA*_A_*) receptors [108,109,110].

**Table 1:** Principal bile acid receptors: distribution, signaling, physiological roles and modulation by bile acid composition.

Receptor (Type)	Major Distribution Cell Types (Human/Rodent)	Canonical Signaling Pathway	Key Physiological Processes Regulated (Examples)	How Bile Acid Composition Modulates Receptor Signaling	Key Endocrine/Immune Mediators Relevant for Gut–brain Signaling
FXR (Farnesoid X receptor)—nuclear receptor	Hepatocytes; ileal enterocytes (incl. some L-cells); cholangiocytes; adipose tissue; kidney; immune cells (macrophages, monocytes, dendritic cells); low-moderate expression in hypothalamus and other brain regions [72,82,111,112].	Ligand (bile acid)-activated nuclear receptor → heterodimer with RXR → transcriptional regulation of SHP, CYP7A1/CYP8B1, bile acid transporters (BSEP, NTCP, OSTα/β), lipogenesis and gluconeogenesis genes; in ileum induces synthesis of enterokine FGF19/FGF15 [82,112].	Bile acid homeostasis (negative feedback on bile acid synthesis); glucose metabolism (hepatic gluconeogenesis, insulin sensitivity); lipid metabolism (tiacylglycerols, cholesterol); appetite and energy balance via FXR-FGF19-hypothalamus axis; immune modulation (innate and adaptive immune cell function) [111,112,113].	Potently activated by CDCA and other primary, relatively hydrophobic bile acids; murine muricholic acids (MCA) act as FXR antagonists, causing interspecies differences. Increased conversion to secondary bile acids (DCA, LCA) reduces FXR agonist activity and shifts signaling toward TGR5; conjugation with glycine or taurine reduces FXR agonistic activity, local pH also modulates bile acids’ protonation state and indirectly FXR activation in intestine vs. liver [82,114,115].	FGF19/FGF15 (enterohepatic endocrine loop to liver and, indirectly, CNS); SHP; downstream changes in insulin signaling; FXR activation in immune cells modulates cytokines (↓ TNF-α, IL-1β; ↑ antimicrobial peptides). These endocrine/immune changes indirectly affect CNS inflammation and energy homeostasis [111,112].
TGR5/GPBAR1—membrane GPCR	Enteroendocrine L-cells (ileum/colon); gallbladder epithelium; brown and white adipocytes; skeletal muscle; cholangiocytes; macrophages and other immune cells; nodose ganglion/vagal afferents; neurons and astrocytes in hippocampus, hypothalamus, cortex and spinal cord [68,72,116].	Gs-coupled GPCR → ↑ cAMP → PKA/CREB, Epac, downstream ion channel modulation; rapid non-genomic effects. In neurons and microglia, modulates excitability and inflammatory signaling [68,117].	Appetite and satiety via GLP-1 and PYY release and direct hypothalamic actions; energy expenditure and thermogenesis (BAT activation, sympathetic outflow); glucose homeostasis (enhanced incretin effect, improved insulin sensitivity); neuroinflammation and neuroprotection (reduced microglial activation, improved synaptic plasticity; roles in neuropathic pain, depression and neurodegeneration) [68,116,118,119]	Highest affinity for secondary, hydrophobic BA (LCA ≈ DCA >> CDCA/CA); thus microbial 7α-dehydroxylation that expands DCA/LCA pool biases signaling toward TGR5. Conjugation reduces membrane permeability but TGR5 is located basolaterally, so conjugated bile acids can still activate it after absorption. Changes in bile acid pool hydrophobicity (diet, microbiota, cholestasis, bariatric surgery) therefore strongly influence TGR5-dependent GLP-1 release, thermogenesis and anti-inflammatory effects [113,116,120].	GLP-1, PYY, OXM from L-cells (gut–brain endocrine loop); catecholamine/sympathetic outputs (BAT); anti-inflammatory cytokines (↑ IL-10, ↓ TNF-α/IL-1β) in macrophages/microglia. These mediators link intestinal TGR5 activation to CNS effects on appetite, mood, neuroinflammation and cardiovascular regulation [68,116,121].
PXR (Pregnane X receptor, NR1I2)—nuclear receptor	Hepatocytes; enterocytes; some brain endothelial cells and neurons (emerging data); immune cells [111,120,122].	Ligand-activated nuclear receptor that regulates xenobiotic-metabolizing enzymes (e.g., CYP3A4), transporters (MDR1, OATP), and interacts with NF-κB and other inflammatory pathways [111,120].	Detoxification and xenobiotic clearance; indirect modulation of bile acid homeostasis (regulation of CYP and transporter expression); immune regulation via suppression of pro-inflammatory signaling; may influence neuroinflammation by controlling CNS exposure to bile acid derivatives and xenobiotics [111,113].	Several bile acids and oxo-bile acid species can act as weak PXR ligands, but microbial bile acid derivatives and co-metabolites (incl. some secondary bile acid and oxysterols) seem particularly relevant. Changes in bile acid composition and microbiota-derived oxo-bile acids therefore alter PXR activation, which in turn adjusts bile acid detoxification and inflammatory tone [111,113].	Induction of CYP3A and phase II enzymes; modulation of cytokines via NF-κB signaling; these changes can reduce systemic and CNS inflammation and alter drug/bile acid exposure to the brain [87,111].
VDR (Vitamin D receptor)—nuclear receptor	Intestinal epithelium; innate and adaptive immune cells; neurons (cortex, amygdala, caudate putamen, and hypothalamus) [72,111,123].	Vitamin-D-responsive nuclear receptor; regulates genes involved in calcium homeostasis, antimicrobial peptides, and immune modulation; secondary bile acids (LCA) and oxysterol derivatives can weakly interact [72,111,124].	Immune homeostasis; neuroprotection and neuronal survival; possible contributions to metabolic control via gut-immune axis [72,87,111,125,126].	LCA its metabolite 3-keto-LCA and LCA amides are the most efficacious bile acid ligands for VDR; bile acid composition that favors these derivatives may subtly modulate VDR-dependent immune and neuroprotective activities [68,111,127,128].	Induction of antimicrobial peptides, T-regulatory phenotypes, and anti-inflammatory cytokines; these immune mediators indirectly shape gut–brain communication and neuroinflammation [111,120,127,129].
GR (Glucocorticoid receptor)—nuclear receptor modulated by bile acids in cholestasis	Broadly expressed: hypothalamus (CRH neurons), pituitary, adrenal, liver, immune cells; bile acids can access hypothalamus in cholestasis [87,130,131].	Ligand-activated transcription factor controlling HPA axis genes (CRH, POMC, ACTH, steroidogenic enzymes) and wide metabolic and inflammatory programs. Bile acids can act directly or indirectly to modulate GR activity [65,75,130].	Stress response and HPA axis; glucose and energy metabolism via cortisol/corticosterone; immune suppression or dysregulation. In cholestasis, aberrant bile acid-GR interaction in hypothalamus contributes to HPA suppression and altered stress/metabolic responses [130,132].	Pathologically elevated, hydrophobic and conjugated bile acids during cholestasis enter the brain, where they can modulate GR in CRH neurons, suppress CRH expression and down-regulate HPA output. Thus, bile acid pool expansion and altered composition in liver disease directly affect neuroendocrine stress signaling [65,75,87,130].	HPA axis hormones (CRH, ACTH, cortisol/corticosterone); downstream metabolic and immune mediators. Bile acid-driven GR modulation links hepatic cholestasis and bile acid overload to central stress circuitry and cognitive/affective symptoms [65,75,87,130,133].
S1PR2 (Sphingosine-1-phosphate receptor 2)—GPCR activated by certain BA	Brain microvascular endothelium (BBB); hepatocytes; cholangiocytes; Kupffer cells; neurons and astrocytes in hippocampus and cortex; immune cells [103,134,135].	G-protein signaling → Rho/ROCK, ERK, AKT and JNK pathways; regulates cytoskeletal dynamics, tight junctions and inflammatory gene expression [82,103,134,136].	BBB integrity and vascular permeability; neuroinflammation (microglial activation, leukocyte recruitment); liver and systemic inflammation; contributes to encephalopathy and neurodegeneration in cholestatic states [103,134,137,138,139].	Certain conjugated bile acids (e.g., taurocholate, taurolithocholate) can act as agonists of S1PR2, especially when their circulating levels are elevated in cholestasis, leading to Rac1-dependent occludin phosphorylation and BBB leakiness. Thus, bile acid pool shifts toward conjugated, hydrophobic species in liver disease drive S1PR2-mediated barrier disruption and CNS inflammation [103,137,139].	Pro-inflammatory mediators: TNF-α, IL-1β, IL-6; chemokines and MMP-9 that promote leukocyte infiltration; microglial activation. These immune mediators couple S1PR2 activation by bile acids to neuroinflammation and cognitive/behavioral changes [103,137,139].

The List of Abbreviations: ACTH—adrenocorticotropic hormone, BAT—brown adipose tissue, BBB—blood–brain barrier, BSEP—bile salt export pump, CA—cholic acid, CDCA—chenodeoxycholic acid, CNS—central nervous system, CREB—cAMP response element-binding protein, CRH—corticotropin-releasing hormone, CYP7A1—cholesterol 7α-hydroxylase, DCA—deoxycholic acid, DHA/LHA—dehydroxylated bile acids (secondary), DPP-4—dipeptidyl peptidase-4, ERK—extracellular signal–regulated kinase, FGF19/FGF15—fibroblast growth factor 19/murine ortholog 15, FGFR4—fibroblast growth factor receptor 4, FXR—farnesoid X receptor, GABA—γ-aminobutyric acid, GLP-1—glucagon-like peptide-1, GPCR—G protein–coupled receptor, GR—glucocorticoid receptor, HPA axis—hypothalamic–pituitary–adrenal axis, IL-1β—interleukin-1 beta, IL-6—interleukin-6, IL-10—interleukin-10, JNK—c-Jun N-terminal kinase, LCA—lithocholic acid, L-cells—enteroendocrine L-cells producing GLP-1, PYY, MCA—muricholic acids, MMP-9—matrix metalloproteinase-9, NF-κB—nuclear factor kappa-light-chain enhancer of activated B cells, NTCP—Na^+^-taurocholate cotransporting polypeptide, OXM—oxyntomodulin, PKA—protein kinase A, PXR—pregnane X receptor, PYY—peptide YY, Rho—Ras homolog, ROCK—Rho-associated protein kinase, RXR—retinoid X receptor, S1PR2—sphingosine-1-phosphate receptor 2, SHP—small heterodimer partner, SNP—single nucleotide polymorphism, TGR5/GPBAR1—Takeda G protein–coupled bile acid receptor 1, TNF-α—tumor necrosis factor-alpha, VDR—vitamin D receptor, WAT—white adipose tissue. ↓—reduced, ↑—increased.

### 6.2. Indirect Bile Acid Signaling to the CNS via the FXR-FGF 15/19 Pathway

In addition to their direct actions within the CNS, bile acids exert indirect effects on brain function through enterohepatic endocrine signaling pathways. Two key mechanisms mediate this cross-talk: the FXR-dependent fibroblast growth factor (FGF)15/19 pathway and the TGR5-dependent glucagon-like peptide-1 (GLP-1) pathway (Figure 7) [72]. 

Activation of FXR in enterocytes of the distal ileum induces the transcription and synthesis of FGF15 (the murine ortholog of human FGF19), an enterokine with potent hormonal properties [140]. Secreted FGF15/19 enters the portal circulation and reaches the liver, where it binds to fibroblast growth factor receptor 4 (FGFR4) in complex with its co-receptor β-Klotho (KLB). Activation of the FGF19–FGFR4–KLB complex triggers a phosphorylation cascade via the c-Jun N-terminal kinase (JNK)–dependent signaling pathway, leading to suppression of hepatic bile acid synthesis through inhibition of the rate-limiting enzyme CYP7A1 [141].

Although the liver represents the primary target organ of FGF15/19, where it regulates bile acid pool size and glucose metabolism, emerging evidence indicates that FGF15/19 also exerts metabolic effects in adipose tissue and the brain [142,143]. Expression of FGFR4 has been detected in the hypothalamus and medial habenular nucleus [144], and circulating FGF15 directly activates FGFRs in hypothalamic agouti-related peptide (AGRP) neurons to regulate peripheral glucose homeostasis. Activation of FGFR signaling silences AGRP/NPY neurons, improving glucose tolerance via autonomic pathways. Moreover, short peptides that selectively inhibit homodimeric FGFR signaling within the hypothalamus improve glucose homeostasis without inducing hypoglycemia [145].

Both intraperitoneal and intracerebroventricular administration of FGF19 in mice suppress hypothalamic AGRP/NPY neuron activity and enhance peripheral glucose metabolism [72]. In obese mice, oral tauroCA gavage increases ileal FGF15 transcription and improves glucose tolerance, with subsequent inhibition of AGRP/NPY neuronal activity through FGFR signaling [145]. Downstream signaling involves FRS1/2, ERK1/2, and STAT3 activation, pathways that overlap with those of the insulin and leptin receptors. However, unlike leptin, FGF19 does not affect POMC gene transcription. Notably, the beneficial metabolic effects of tauroCA are abolished in mice lacking FGFR1 specifically in AGRP/NPY neurons, indicating that hypothalamic FGFR1 is required for mediating bile acid–induced improvements in glucose tolerance [145].

A key unresolved question is whether physiologically relevant postprandial increases in circulating FGF15/19 are sufficient to exert measurable effects on CNS activity [72]. Centrally administered FGF19 improves glucose tolerance and insulin sensitivity independently of body weight or peripheral insulin signaling, effects that may involve suppression of the HPA axis [107,146]. In individuals with metabolic syndrome or obesity, circulating FGF19 levels are reduced, whereas CYP7A1 expression and total bile acid pool size are increased in type 2 diabetes, suggesting disruption of the negative FXR/FGF19 feedback loop [147].

Despite the metabolic benefits of bile acid-driven FGF15/19 signaling, chronic suppression of bile acid synthesis poses potential risks. As bile acid biosynthesis represents the principal pathway for cholesterol catabolism, long-term inhibition—whether through bile acid supplementation or FGF15/19 therapy, may promote cholesterol accumulation and increase cardiovascular risk. Furthermore, sustained FGF19 overexpression in mice induces hepatocellular carcinoma, primarily via STAT3 phosphorylation and constitutive activation of hepatic FGFR4 [148].

### 6.3. Bile Acid Signaling to the CNS via the TGR5-GLP-1 Pathway

Enteroendocrine L cells, which secrete glucagon-like peptide-1 (GLP-1), express a wide array of receptors responsive to metabolites derived from the intestinal microbiota, including GABA, SCFAs, and 5-HT [149]. In isolated human enterocytes, approximately 75% of GLP-1-expressing cells express TGR5, whereas only 16% of GLP-1-negative cells do so, indicating that L cells are the predominant intestinal cell type responsive to TGR5 activation [150]. The TGR5 receptor is primarily activated by secondary bile acids, whose concentration and composition are determined by gut microbial activity. Recent evidence demonstrates that specific gut microbial taxa and their enzymatic activities are responsible for the biotransformation of primary bile acids into a diverse pool of secondary bile acids with distinct receptor affinities and signaling properties. The gut microbiota not only contributes to the generation of deconjugated bile acids but also plays a pivotal role in converting bile acids into a variety of downstream metabolites. In humans, four major microbial transformations of bile acids have been characterized—deconjugation, dehydroxylation, oxidation, and epimerization, all of which have been comprehensively reviewed in the recent literature [151,152]. A bidirectional regulatory interplay exists between the gut microbiota and bile acid pool. Microbial communities modulate bile acid metabolism, thereby shaping both the size and the chemical diversity of the bile acids pool. Conversely, through their distinct physicochemical properties and physiological activities, bile acids further exert selective effects on intestinal microorganisms, promoting or inhibiting the growth of specific taxa and ultimately influencing the overall structure of the gut microbial community. Bacteria expressing bile salt hydrolase (BSH)—including species from the genera *Bacteroides*, *Lactobacillus*, *Bifidobacterium*, *Enterococcus*, and *Clostridium*—catalyze the initial deconjugation step that enables further microbial modification of primary bile acids (Figure 4 and Figure 5, Table 2) [153,154]. The most potent 7α-dehydroxylating activity is mediated by *Clostridium* cluster XIVa and XI species, particularly *Clostridium scindens*, *C. hylemonae*, and *C. hiranonis*, which express the *bai* operon (BaiCD, BaiE, BaiA) and convert cholic and chenodeoxycholic acids into the secondary bile acids deoxycholic acid (DCA) and lithocholic acid (LCA), both strong agonists of TGR5 and modulators of FXR signaling [155,156]. Additional transformations, including oxidation and epimerization of hydroxyl groups at positions C3, C7 and C12, mediated by microbial hydroxysteroid dehydrogenases (HSDHs) from genera such as *Eubacterium*, *Ruminococcus* and *Bacteroides*, further diversify the bile acid pool and its receptor pharmacology [82]. These microbial transformations act synergistically with other microbiota-derived metabolites to regulate bile acid receptor activity in the gut and central nervous system. SCFAs—acetate, propionate, butyrate, produced by fibre-fermenting taxa (e.g., *Faecalibacterium*, *Roseburia*, *Anaerostipes*), activate FFAR2/3 on enteroendocrine L cells, stimulate GLP-1 and PYY secretion, and upregulate intestinal expression of both TGR5 and FXR, thereby increasing sensitivity to secondary bile acid signaling [157,158]. Tryptophan-derived indoles produced by certain *Lactobacillus* and *Clostridium* species act through the aryl hydrocarbon receptor (AhR) to modulate inflammation and barrier function, indirectly potentiating TGR5-mediated responses [159]. In parallel, SCFAs and secondary bile acids stimulate enterochromaffin cells to release serotonin, which, although unable to cross the BBB, activates vagal afferents and complements the GLP-1-TGR5 axis in gut–brain communication [160,161]. Collectively, these metabolites form an integrated, synergistic network that enhances receptor expression, increases ligand availability, and converges on common neuroendocrine circuits, including vagal afferents and hypothalamic pathways, thereby exerting a coordinated regulatory effect on the gut–brain axis (126). The activation of TGR5 enhances glucose homeostasis by stimulating GLP-1 secretion from L cells (118). In addition, L cells express FXR, which also modulates GLP-1 synthesis (119) Since TGR5 is localized on the basolateral membrane of L cells, bile acids must traverse the intestinal epithelium to activate the receptor and induce GLP-1 release [162].

Alternative, TGR5-independent pathways can also trigger GLP-1 secretion. Although TGR5-null mice are capable of producing GLP-1, they exhibit impaired glucose tolerance when fed a high-fat diet, suggesting that TGR5-mediated signaling becomes particularly important under metabolic stress [81]. In contrast to the stimulatory effects of secondary and conjugated bile acids on the TGR5-GLP-1 signaling axis, primary bile acids preferentially activate FXR, which suppresses GLP-1 secretion. Conversely, intestinal FXR deletion enhances GLP-1 levels [163]. Crosstalk between FXR and TGR5 contributes to the fine-tuning of glucose homeostasis [164]. These receptors can also exert opposing effects on autophagy: activation of FXR (primarily by primary bile acids) inhibits autophagy [165] whereas activation of TGR5 (by secondary bile acids) promotes autophagy [166].

Beyond the intestine, TGR5 activation exerts systemic metabolic effects. In the liver and brown adipose tissue, TGR5 signaling promotes anti-inflammatory responses and enhances energy expenditure, respectively [167]. Through binding of GLP-1 to its receptor on pancreatic β-cells, postprandial insulin secretion is stimulated, improving glucose tolerance and promoting hepatic glycogen synthesis. Circulating GLP-1 can also cross the BBB and act directly in the CNS, where it binds to neuronal GLP-1 receptors to suppress appetite and enhance satiety [168]. The GLP-1 receptor is also expressed in vagal afferent neurons of the gastrointestinal tract. Because the enzyme dipeptidyl peptidase-4 (DPP-4) rapidly degrades GLP-1 in the plasma and capillary endothelium, it is likely that bile acid-induced GLP-1 signaling is transmitted to the CNS predominantly via vagal afferents rather than through circulating GLP-1 [169]. Indeed, GLP-1 signaling through vagal afferents modulates feeding behavior, energy expenditure, glucose metabolism, and cardiovascular function [170].

Expression of TGR5 within the brain further suggests that bile acids may act as direct gut-derived neuroactive signals [84]. Activation of central TGR5 triggers anti-inflammatory responses: administration of tauroursodeoxycholic acid (TUDCA) in models of acute neuroinflammation induces interleukin-10 (IL-10) expression, suppresses vascular cell adhesion molecule-1 (VCAM-1) and monocyte chemoattractant protein-1 (MCP-1) expression, and inhibits microglial migration in a TGR5-dependent manner [171,172]. Similarly, in a mouse model of hepatic encephalopathy, the TGR5 agonist betulinic acid reduced cortical levels of IL-1, IL-6, and tumor necrosis factor-α (TNF-α), concomitant with reduced microglial activation [98].

**Table 2:** Reactions of the biotransformation of bile acids by intestinal microbiota.

Type of Modification	Enzyme Catalyzing Biotransformation Reaction	Bacteria	Site of Action	Reaction	Product	Ref.
Deconjugation	Bile salt hydrolase (BSH)	*Actinobacteria*, *Turicibacter*, *Bacteroides*, *Lactobacillus*, *Parabacteroides*, *Bifidobacterium*, *Clostridium*, *Enterococcus*, *Listeria*, *Stenotrophomonas*, *Brucella*	C24	-COO-Gly/Tau-COOH	Tauro/Glyco CA → CA Tauro/Glyco CDCA → CDCA	[173,174,175]
Dehydroxylation	*bai* operon	*Clostridium*, *Eubacterium*, *Lachnospiraceae*, *Ruminicoccaceae*, *Peptostreptococcaceae*	C7	-OH → -H	CA → DCA CDCA → LCA CDCA → UDCA → LCA	[176,177]
Oxidation and epimerization	3 α/β Hydroxysteroid dehydrogenase	*Parabacteroides merdae*, *Odoribacteriaceae*, *Ruminococcus gnavus*, *Blautia producta*, *Eggerthella genus*, *Enterorhabdus mucosicola*, *Acinetobacter lwoffii*	C3	α/β-OH ↔ =O	CA → 3-oxo-CA → Iso-CA CDCA → 3-oxo-CDCA → Iso-CDCA LCA → 3-oxo-LCA → Iso-LCA	[178,179,180]
	7 α/β Hydroxysteroid dehydrogenase	*Clostridium baratii Ruminococcus gnavus*, *Clostridium absonum*, *Stenotrophomonas maltophilia*, *Collinsella aerofaciens*	C7	α/β-OH ↔ =O	CDCA → 7-oxo-LCA → UDCA CA → 7-oxo-DCA → UCA	[181,182]
	12 α/β Hydroxysteroid dehydrogenase	*Eggerthella lenta*, *Enterorhabdus mucosicola*, *Clostridium scindens*, *Peptacetopacter hiranonis*, *Clostridium hylemonae*, *Bacteroides*, *Clostridium paraputrificium*, *Clostridium tertium*, *Clostridium difficile*	C12	α/β-OH ↔ =O	CA → 12-oxo-CDCA → epi-CA	[82,183,184]

List of Abbreviations: BA—bile acids, BSH—bile salt hydrolase, CA—cholic acid, CDCA—chenodeoxycholic acid, DCA—deoxycholic acid, LCA—lithocholic acid, UDCA—ursodeoxycholic acid, UCA—ursodeoxycholic acid epimer (ursodeoxycholate), C24—carbon-24 position of the steroid backbone, C3/C7/C12—carbon positions at which oxidation, epimerization or hydroxyl modifications occur, bai operon—bile acid–inducible gene cluster responsible for 7α-dehydroxylation, HSDH—hydroxysteroid dehydrogenase (3α/β-HSDH, 7α/β-HSDH, 12α/β-HSDH), -OH—hydroxyl group, =O—keto group, Gly/Tau—glycine- and taurine-conjugated bile acids, Iso-BA—iso-bile acid (epimer formed by 3- or 7-epimerization).

## 7. Intestinal Microbiota–Enteroendocrine/Enterochromaffin Cell Axis

The gut microbiota profoundly influences gut–brain communication through the production of numerous microbial metabolites (Table 3). These metabolites act as crucial signaling molecules mediating host–microbe interactions via enteroendocrine cells and enterochromaffin cells. SCFAs are generated by the microbial fermentation of dietary-resistant starches and non-starch polysaccharides and play multiple physiological roles, including regulation of host energy metabolism, stimulation of colonic blood flow, enhancement of fluid and electrolyte absorption, and promotion of mucosal growth.

SCFAs are predominantly produced in the distal gastrointestinal tract, with butyrate, propionate, and acetate representing approximately 90–95% of total SCFAs in the colon [185]. Among these, butyrate serves as the primary energy source for colonocytes; propionate is transported via the portal vein to the liver, where it supports gluconeogenesis; and acetate enters systemic circulation to act on peripheral tissues [186]. SCFAs modulate the secretion of gut peptides, notably GLP-1, through activation of free fatty acid receptors FFAR2 (GPR43) and FFAR3 (GPR41) expressed on L cells [187]. In primary murine intestinal cultures, SCFAs increase GLP-1 secretion, whereas this effect is absent in FFAR2^−/−^ and FFAR3^−/−^ cultures and markedly reduced in knockout mice, underscoring the receptor dependence of this pathway [187]. Furthermore, prebiotic interventions enhance the differentiation of enteroendocrine cells and increase gut peptide synthesis, reinforcing the role of microbial metabolites in enteroendocrine signaling [188].

Preclinical and clinical studies demonstrate that microbial activity, particularly SCFA production stimulates L cells in the distal ileum to release peptide YY and GLP-1, thereby inducing satiety and modifying feeding behavior [19]. Notably, FFAR3 is also expressed in postganglionic sympathetic and sensory neurons within both the autonomic and somatic peripheral nervous systems, suggesting that SCFAs modulate gut–brain communication not only through enteroendocrine signaling but also by directly influencing neural circuits that integrate gastrointestinal and autonomic function [35].

A second major component of this microbiota–gut–brain axis involves 5-HT, which is synthesized predominantly by intestinal enterochromaffin cells. Approximately 95% of the body’s total serotonin is stored within enterochromaffin cells and enteric neurons, with only 5% localized in the CNS. Gut-derived serotonin is secreted in response to luminal nutrients and acts locally on vagal afferent fibers expressing diverse serotonin receptor subtypes, including 5-HT_2_ and 5-HT_4_ receptors, which are also distributed throughout the CNS, gastrointestinal tract, heart, and adrenal glands [189].

Activation of 5-HT receptors suppress appetite and reduces body weight, whereas pharmacological antagonism of these receptors produces hyperphagia and weight gain [190]. Metabolomic profiling of germ-free mice reveals a greater than twofold reduction in circulating 5-HT levels compared with conventionally colonized counterparts [191]. Restoration of bile acid metabolism, specifically the microbial conversion of cholic acid to deoxycholic acid, correlates with increased colonic and serum 5-HT concentrations and elevated expression of tryptophan hydroxylase 1, the rate-limiting enzyme in peripheral serotonin biosynthesis [18].

Both SCFAs and secondary bile acids produced by spore-forming intestinal bacteria regulate a substantial portion of serotonin synthesis and release from enterochromaffin cells [192]. Interestingly, inhibition of peripheral serotonin synthesis has been shown to ameliorate obesity and metabolic dysfunction, as serotonin suppresses β-adrenergic signaling in brown adipose tissue, thereby reducing thermogenesis [193].

Recent findings have identified TGR5-expressing enterochromaffin cells in the colon, but not in the small intestine, that release serotonin in response to bile salt stimulation [194]. Moreover, neoplastic enterochromaffin cells display heightened sensitivity to bile acid ligands [195]. Although serotonin cannot cross the BBB, enterochromaffin cells form synapse-like contacts with sensory nerve fibers, establishing a direct neuroepithelial communication pathway through which luminal signals can influence central neural activity [196].

Although enterochromaffin cells and their serotonin output represent a central hub in gut–brain communication, enteroendocrine and enterochromaffin cells are also exposed to, and respond to, a broader spectrum of microbiota-derived neurotransmitters, including GABA, dopamine (DA) and norepinephrine (NE). These signals act in parallel with serotonin to shape hormone secretion, vagal afferent activity and local immune tone, thereby contributing to a more complex and integrated intestinal microbiota–enteroendocrine/enterochromaffin cell axis [197,198,199]. Multiple gut commensals, particularly lactic acid bacteria such as *Lactobacillus* and *Bifidobacterium* genera, express glutamate decarboxylase (GAD) and convert luminal glutamate into GABA [200]. GABA produced in the intestinal lumen can act locally on GABA*_A_* and GABA*_B_* receptors expressed on intrinsic enteric neurons, vagal afferents and, to a lesser extent, on subsets of enteroendocrine cells, modulating motility, visceral sensitivity and peptide release [201,202]. Experimental and translational studies suggest that GABA-producing strains can increase GLP-1 and PYY secretion, likely via paracrine activation of enteroendocrine cells and modulation of enteric reflex circuits, with downstream effects on appetite control, glucose tolerance and stress responsivity [200,203]. In parallel, GABAergic signaling along vagal afferents and within the NTS has been implicated in the anxiolytic and antidepressant-like effects of “psychobiotic” strains in preclinical models, providing a mechanistic link between microbial GABA production, enteroendocrine/enterochromaffin function and central emotional circuits [85,204]. A range of gut bacteria, including *Bacillus*, *Escherichia*, *Staphylococcus* and *Proteus* species, can synthesize DA and NE from aromatic amino acid precursors, and germ-free or antibiotic-treated animals show marked alterations in luminal and tissue catecholamine concentrations [205,206]. Microbiota-derived catecholamines, together with host-derived NE from sympathetic fibers, act on adrenergic and dopaminergic receptors expressed by enteroendocrine/enterochromaffin cells, immune cells and enteric neurons [205,206]. In enterochromaffin cells, DA and NE modulate 5-HT release and mechanosensory responsiveness, thereby indirectly influencing peristalsis, secretion and nociception [198,207]. In enteroendocrine cells, catecholaminergic signaling integrates with nutrient-sensing pathways to modulate the secretion of ghrelin, CCK, and GLP-1. Notably, sympathetic norepinephrine can attenuate GLP-1 release under specific physiological conditions, thereby functionally coupling central autonomic output to peripheral gut hormone regulation [208]. At the level of the microbiota—immune—neural interface, DA and NE not only act as neuromodulators but also influence bacterial growth and virulence, creating bidirectional feedback between catecholaminergic tone and microbial composition [209,210]. Stress-related increases in intestinal catecholamines have been linked with shifts in microbial composition, increased permeability and altered enteroendocrine/enterochromaffin cells activation patterns, potentially contributing to comorbid anxiety, depression, functional gastrointestinal disorders and cardiometabolic disease [211]. Recent reviews further emphasize that microbiota-dependent modulation of DA and NE in the gut can alter central catecholaminergic signaling via vagal afferent pathways, immune mediators and HPA axis regulation, even though these monoamines themselves do not efficiently cross the BBB [212]. Therefore, microbiota-derived GABA, DA and NE add additional layers of regulation to the serotonin-centered enterochromaffin axis. enteroendocrine and enterochromaffin cells function as multimodal sensors that integrate microbial metabolites (SCFAs, secondary bile acids, indoles), classical neurotransmitters and immune signals to shape gut hormone release and afferent neural signaling [213]. Through their effects on GLP-1, PYY, ghrelin and 5-HT, as well as on vagal and spinal afferents, GABA and catecholamines contribute to the control of appetite, energy balance, mood and pain perception, and may modulate susceptibility to metabolic and neuropsychiatric disorders [85,214]. Dysbiosis is usually defined as a disturbance of the normal gut microbial ecosystem, involving alterations in community structure, taxonomic composition, and functional activity [215,216]. These alterations may result from diet, medication, chronic disease, lifestyle or environmental factors. However, given the wide interindividual variability in gut microbiota composition, there is currently no gold standard for a “healthy” microbiota baseline. Accordingly, the characterization of dysbiosis relies on tools that assess gut microbial composition and function. Microbiota composition is most commonly assessed using 16S rRNA gene sequencing, which provides bacterial taxonomic profiles, while shotgun metagenomics provides in-depth analysis of microbial composition together with functional gene content. Advances in sample preservation also allow emerging tools such as RNA-based profiling (metatranscriptomics), which may identify the metabolically active fraction of the microbiota and is increasingly used to detect physiologically relevant dysbiosis [217]. Disturbance in microbial bile acid-modifying activity (reflected as changes in secondary-to-primary bile acid ratios) is emerging as a relevant biomarker of gut microbiota dysbiosis with potential systemic and neuro-metabolic repercussions [218,219]. Consequently, dysbiosis-induced disturbances in bile acid signaling represent a mechanistic link between microbial imbalance and CNS-related metabolic or neuropsychiatric pathology, further supported by emerging human studies evidence [220,221,222,223,224].

**Table 3:** Microbiota-derived metabolites that modify bile acid metabolism and signaling, and their impact on CNS and metabolic disease.

Metabolite Class (Examples)	Predominant Microbial Sources/Enzymes	Effect on Bile Acid Metabolism and Pool Composition	Mechanism Altering Bile Acid Signaling (Receptors/Pathways)	Impact on CNS/Metabolic/Disease Processes	Ref.
Short-chain fatty acids (SCFAs) (acetate, propionate, butyrate)	Produced by *Firmicutes* (e.g., *Faecalibacterium*, *Roseburia*), *Bacteroidetes*, via fermentation of dietary fibers and resistant starch.	Indirectly modulate bile acid synthesis by regulating hepatic cholesterol metabolism and FXR-FGF19 axis; can influence bile acid pool size and proportion of conjugated vs. unconjugated bile acids through effects on hepatic and intestinal gene expression; may change microbiota composition favoring/de-favoring bile acid-transforming taxa	Activate FFAR2/FFAR3 on enteroendocrine L cells, enhancing GLP-1/PYY secretion and thereby crosstalking with bile acid-TGR5-FXR signaling; SCFAs also modulate intestinal barrier and systemic inflammation, which impact BA receptor sensitivity, epigenetic effects (HDAC inhibition) alter host gene expression (including bile acid synthesis genes); indirectly upregulate TGR5/FXR expression in gut.	Improve glucose homeostasis, insulin sensitivity and body weight in preclinical and human studies; SCFA–EEC signaling contributes to gut–brain regulation of appetite and may influence neuroinflammation and cognitive function via GLP-1 and vagal pathways.	[157,214,225,226,227,228]
BSH-mediated deconjugation products (unconjugated primary BA)	Gut bacteria expressing bile salt hydrolase (BSH) (e.g., *Lactobacillus*, *Bifidobacterium*, *Bacteroides*, *Clostridium*).	Hydrolyze glycine/taurine-conjugated bile acids to free bile acid species, increasing hydrophobicity and availability for further microbial transformations (e.g., 7α-dehydroxylation); reshape ratio of conjugated/unconjugated bile acids in ileum and colon.	Deconjugation alters affinity for FXR and TGR5 (unconjugated species often more hydrophobic, with different receptor potency); changes intestinal FXR tone and downstream FGF19 signaling; modifies bile acids reabsorption kinetics.	Implicated in modulation of MASLD/MASH, cholesterol homeostasis and glucose metabolism; BSH-active probiotics can lower cholesterol and alter bile acid signaling; altered BSH profiles associate with metabolic syndrome and chronic liver disease; may modulate BBB permeability indirectly via bile acid species shifts.	[229,230,231,232]
7α-dehydroxylation products (secondary BA) (deoxycholic acid, DCA; lithocholic acid, LCA)	Low-abundance 7α-dehydroxylating *Clostridia* (e.g., *Clostridium scindens*), expressing bai gene cluster (BaiA–BaiI).	Convert primary bile acids (CA, CDCA) to hydrophobic secondary bile acids (DCA, LCA), substantially increasing the fraction of potent TGR5 agonists and altering the primary/secondary bile acids ratio.	DCA and LCA are high-affinity TGR5 ligands and can also modulate FXR; enhanced 7α-dehydroxylation shifts signaling from ileal FXR-FGF19 toward TGR5 in intestine, adipose tissue and possibly CNS; increased hydrophobic bile acids may cross BBB more readily, affecting neuroinflammation.	Support GLP-1-mediated improvement in energy expenditure and glucose metabolism, but excess hydrophobic bile acids are associated with mucosal injury, colon cancer risk and liver injury; recent studies links 7α-dehydroxylating strains to mucosal healing and bile acid homeostasis in colitis.	[155,233,234,235]
Microbially conjugated bile acids (microbial bile acid amides MABAs) (e.g., Phe-CA, Leu-CA, Trp-CA)	Diverse human gut microbiota re-conjugating bile acids with amino acids (phenylalanine, leucine, tyrosine, tryptophan, branched-chain and non-proteinogenic amino acids).	Generate novel bile acid species (MABAs) with distinct hydrophobicity and receptor affinity; expand bile acid chemical repertoire beyond classical taurine/glycine conjugates; some species are enriched or depleted in metabolic disease (e.g., Trp-CA ↓ in T2D).	Several MABAs directly activate TGR5 and both intestinal and hepatic FXR isoforms, thereby modulating GLP-1 secretion, FGF19 signaling and hepatic bile acid synthesis; specific conjugates (e.g., Trp-CA) improve glucose tolerance in vivo.	Altered MABA profiles correlate with T2D, obesity and inflammatory bowel disease; experimental data indicate improved glucose homeostasis and reduced adiposity with specific MABAs; potential to modulate gut–brain signaling via GLP-1 and FGF19.	[236,237,238,239,240,241]
Hydroxylated and oxidized bile acid species (e.g., 6α-hydroxylated bile acids, oxo-bile acids)	Formation influenced by diet-responsive microbiota and host–microbial enzymes (hydroxylases, dehydrogenases); fiber-enriched microbiota can increase 6α-hydroxylated bile acids.	Modify bile acid pool toward more hydrophilic species with selective receptor profiles; 6α-hydroxylated bile acids partially replace classical secondary bile acids in response to prebiotic/fiber interventions.	6α-hydroxylated bile acids are potent TGR5 agonists that enhance GLP-1 release and energy expenditure; some oxo-bile acids act as partial FXR agonists/antagonists, fine-tuning FXR signaling in intestine and liver.	In murine models, 6α-hydroxylated bile acids improve glucose metabolism and body weight via TGR5; observational data suggest links between altered hydroxylated bile acid profiles, insulin resistance and cardiometabolic risk; potential indirect effects on CNS through improved metabolic control and reduced inflammation.	[242,243,244,245,246]
Tryptophan metabolites (indoles: IPA, IAA, IAld)	*Lactobacillus*, *Clostridium*, *Peptostreptococcus*, *Bacteroides*	Do not directly change bile acid chemical structures but modulate host inflammation and barrier function, thereby altering microbiota composition and downstream bile acid transformations (indirect reshaping).	Activate AhR and PXR in intestinal and immune cells → strengthen barrier, modulate CYP-mediated bile acid metabolism; reduce inflammation that otherwise perturbs bile acid processing.	Improve barrier integrity, reduce intestinal inflammation, indirectly favor beneficial bile acid profiles; implicated in MASLD, metabolic disease and neuroimmune regulation.	[247,248,249]
Integrated microbiota– bile acid metabolite networks (SCFAs, secondary BA, indoles)	Complex consortia of gut microbes producing SCFAs, secondary bile acids and tryptophan-derived indoles.	Coordinate regulation of bile acid synthesis, conjugation and transformation; shape bile acid pool composition and distribution along the intestine.	SCFAs → FFAR2/3; secondary bile acids and MABAs → FXR/TGR5; indoles → AhR and intestinal barrier regulation; combined effects converge on GLP-1, FGF19, inflammatory cytokines and vagal signaling, integrating metabolic and gut–brain pathways	Dysregulated metabolite networks are associated with obesity, T2D, MASLD/MASH, IBD and neuropsychiatric/neurodegenerative disorders; balanced profiles correlate with healthier metabolic and cognitive phenotypes and preserved gut–brain homeostasis.	[225,250,251,252,253]

The List of Abbreviations: AhR—aryl hydrocarbon receptor, BA—bile acids, BBB—blood–brain barrier, Bai genes (BaiA–BaiI)—bile acid–inducible gene cluster responsible for 7α-dehydroxylation, BSH—bile salt hydrolase, CA—cholic acid, CDCA—chenodeoxycholic acid, CNS—central nervous system, DCA—deoxycholic acid, EEC—enteroendocrine cell, FFAR2/FFAR3—free fatty acid receptor 2/3, FGF19/FGF15—fibroblast growth factor 19/15, FXR—farnesoid X receptor, GLP-1—glucagon-like peptide-1, HDAC—histone deacetylase, IAA—indole-3-acetic acid, IAld—indole-3-aldehyde, IPA—indole-3-propionic acid, IBD—inflammatory bowel disease, LCA—lithocholic acid, MASLD/MASH—metabolic dysfunction–associated steatotic liver disease/metabolic dysfunction–associated steatohepatitis, MCBAs/MABAs—microbially conjugated bile acids (microbial bile acid amides), PXR—pregnane X receptor, PYY—peptide YY, SCFAs—short-chain fatty acids, T2D—type 2 diabetes, TGR5 (GPBAR1)—Takeda G-protein–coupled bile acid receptor 1, Trp-CA—tryptophan-conjugated cholic acid.

## 8. Microbiota–Gut–Brain Axis in Neurodegeneration

The microbiota–gut–brain axis plays a significant role in the progression of neurodegenerative diseases such as Alzheimer’s and Parkinson’s disease, where disruptions in gut microbiota composition may impact CNS homeostasis through immune, neural, endocrine, and metabolic pathways. Dysregulation of the microbiota–gut–brain axis contributes to neuroinflammation, BBB disruption, protein misfolding and aggregation, and impaired neurotransmitter regulation, eventually altering neuronal survival and promoting the pathogenesis of neurodegenerative disorders [254,255]. Microbial metabolites interact extensively with host physiological systems, emphasizing the significance of the gut microbiota in modulating neurodegenerative processes.

Gut dysbiosis has been increasingly recognized as a factor influencing BBB integrity. The BBB maintains the microenvironment of the brain by regulating nutrient transport and restricting the passage of toxins and immune cells. Dysbiosis can compromise BBB structure through inflammatory cytokine release, harmful microbial metabolites, and reduced production of protective SCFAs. Increased permeability allows neurotoxic substances and peripheral inflammatory mediators to enter the CNS and further promote neurodegenerative progression [224,256,257]. Lipidomic, proteomic, and metabolomic studies of BBB endothelial cells highlight how microbial signals impact the membrane composition, tight junction stability, and cellular metabolism, leading to barrier dysfunction [134].

Neuroinflammation is one of the most prominent consequences of microbiota–gut–brain axis disruption. Dysbiosis can induce systemic inflammation characterized by increased pro-inflammatory cytokine levels, such as IL-6, and activation of immune pathways such as NF-κB signaling [254,255]. These cytokines weaken tight junction function and increase BBB permeability [258]. Additional harmful metabolites produced by dysbiotic microbiota, such as p-cresol sulfate, further impair BBB integrity by activating signaling pathways that degrade tight junction proteins [259].

In addition to neuroinflammation, microbiota–gut–brain axis disruption contributes to the misfolding and aggregation of proteins central to neurodegenerative diseases. Metabolites like trimethylamine N-oxide and lipopolysaccharides promote aggregation of amyloid-β, tau, and α-synuclein, thereby exacerbating Alzheimer’s and Parkinson’s pathology [224,255]. Dysbiosis also influences neurotransmitter production and neuroactive compounds that regulate cognition and motor function. Disturbances in these microbial pathways are linked to neurodegenerative symptomatology [256,260].

Impairments in BBB function further amplify these effects. Reduced SCFA production resulting from gut dysbiosis weakens BBB tight junctions, as SCFAs normally upregulate proteins such as occludin, claudin-5, and zona occludens-1 while simultaneously modulating inflammatory pathways [261]. Reduced levels of SCFA-producing bacteria during dysbiosis facilitate the influx of neurotoxic metabolites and immune cells into the CNS, thereby promoting neuroinflammation and cognitive decline [258,262]. Antibiotic-induced depletion of gut microbiota has been demonstrated to decrease tight junction protein expression and increase BBB permeability, while restoration of normal microbial communities reverses these effects [263].

These mechanisms underscore the therapeutic potential of targeting the microbiota–gut–brain axis to mitigate neurodegeneration. Probiotics and prebiotics have shown promise in restoring microbial balance, reducing inflammation, and improving cognitive and motor outcomes in preclinical studies [264,265]. Fecal microbiota transplantation is also being investigated for its ability to restore the healthy microbiota composition and modulate microbiota–gut–brain axis pathways, with certain findings suggesting improvements in metabolic and neurological parameters [254,256]. Dietary interventions aimed at increasing SCFA-producing bacteria and reducing dysbiosis-associated metabolites have similar potential in slowing neurodegenerative disease progression [266,267].

Emerging microbiome-based therapies, including targeted microbial consortia, engineered probiotics, and metabolite-directed interventions, offer additional options for modulating activity of microbiota–gut–brain axis, with ongoing research focusing on personalized treatment strategies and the development of biomarkers for better therapeutic targeting [254,268]. Despite promising findings and developments, significant challenges remain. Individual variability in microbiota composition, incomplete understanding of causal pathways, and complexity of host–microbe interactions limit the clinical translation. Therefore, further mechanistic and longitudinal studies are needed to clarify these relationships and optimize therapeutic strategies for neurodegenerative diseases. However, it is clear that microbiota–gut–brain axis represents a critical intersection between gut microbial dynamics, BBB integrity, systemic inflammation, and neurodegenerative pathology. Understanding and therapeutically targeting this axis hold real promise for improving outcomes in Alzheimer’s disease, Parkinson’s disease, and other neurodegenerative disorders.

## 9. Summary of Clinical Evidence

Most mechanistic insights into bile acid-mediated gut–brain communication have been derived from rodent models; however, a growing body of clinical and translational work has begun to delineate how these pathways operate in humans. Overall, human data support a role for bile acids as integrators of metabolic, neuroendocrine and neurocognitive function, but the magnitude and consistency of effects are generally more modest and heterogeneous than those observed in inbred rodent strains [269,270].

In animal models, manipulation of bile acid pools and FXR/TGR5 signaling produces large and reproducible improvements in glucose tolerance, energy expenditure and adiposity. These findings are partly recapitulated in humans undergoing bariatric surgery, where multiple cohorts show that rises in circulating bile acids, particularly postprandial conjugated and secondary species, are associated with augmented GLP-1 and PYY responses and improved glycaemic control [167,271,272]. Human clinical and metabolomic studies repeatedly show that postprandial and fasting circulating bile acid profiles correlate with insulin sensitivity, glucose tolerance and body composition, and that perturbations in the FXR-FGF19 axis associate with metabolic dysfunction. Reduced circulating FGF19, altered conjugation patterns and expanded bile acid pool size have been linked to insulin resistance and type 2 diabetes in recent cohorts, supporting translational relevance of the FXR-FGF19 feedback loop identified in rodents [273]. Recent longitudinal studies have characterized dynamic changes in the human bile acid metabolome after Roux-en-Y gastric bypass and sleeve gastrectomy, demonstrating that increased postprandial bile acid excursions and altered FGF19 and GLP-1 profiles track with weight loss and insulin sensitivity but exhibit considerable interindividual variability, likely reflecting differences in microbiota, bile acid transporter expression and diet [271,272,274]. These human data are broadly consistent with rodent studies showing that TGR5-mediated GLP-1 release and intestinal FXR–FGF15/19 signaling contribute to the metabolic benefits of bariatric surgery, but also indicate that the effect sizes in clinical populations are smaller and modulated by host and environmental factors [275,276]. Pharmacological activation of bile acid receptors has likewise entered clinical evaluation. Steroidal and non-steroidal FXR agonists (e.g., obeticholic acid and newer intestine-biased FXR agonists) consistently improve metabolic dysfunction-associated steatohepatitis histology and reduce liver fibrosis in phase 2 and 3 trials, but their effects on body weight, insulin resistance and systemic inflammatory markers are more modest than anticipated from preclinical studies [269,277,278]. These trials support FXR as a therapeutic target in metabolic liver disease, yet highlight important differences between rodent and human responses, including pruritus, lipid changes and variable extrahepatic metabolic benefits. Human data on selective TGR5 agonists remain limited, in part due to concerns about gallbladder and off-target effects, so most evidence for TGR5-mediated metabolic and neuroprotective actions still derives from animal models [12,269]. Clinical interest in modulating the FXR-FGF19 axis has intensified. Recent reviews and early human studies summarize that restoring or mimicking FGF19 signaling can improve metabolic parameters, but long-term safety concerns remain (notably mitogenic risks observed with FGF19 overexpression in animal models) [279]. Thus, while human studies data support metabolic effects of FGF19 modulation, therapeutic development requires careful dose-finding and safety monitoring [280].

Diet, antibiotic use, and probiotics dynamically shape the composition and size of the bile acid pool, influencing both the relative abundance of primary and secondary bile acids and their signaling via FXR and TGR5. Dietary composition modulates bile acid synthesis and conjugation, while antibiotics can disrupt microbial communities, reducing bile acid deconjugation and transformation. Probiotics can restore microbial balance, enhance secondary bile acid production, and indirectly modulate gut–brain signaling. Together, these factors demonstrate the dynamic interplay between gut microbiota, bile acid metabolism, and CNS-mediated regulation of metabolism, appetite and energy homeostasis [281,282,283]. It should be noted that while probiotics, prebiotics, and fecal microbiota transplantation generally modulate bile acid signaling within physiological ranges, bile acid analogs and selective FXR/TGR5 agonists or antagonists, as most commonly used in preclinical and clinical trials, exert pharmacological effects exceeding normal endogenous concentrations. Table 4 summarizes potential therapeutic strategies targeting bile acid signaling and gut microbiota, including probiotics, prebiotics, fecal microbiota transplantation, bile acid analogs, and selective FXR/TGR5 agonists and antagonists. 

**Table 4:** Modulation of bile acid-dependent signaling: therapeutic interventions, CNS outcomes, and limitations.

Therapeutic Strategy	Mechanism of Action	Impact on Bile Acid Metabolism/Receptors (FXR, TGR5)	Evidence (Preclinical/Clinical)	CNS and Metabolic Effects	Challenges and Limitations	Ref.
Probiotics	Modulate gut microbiota composition, enhance bile salt hydrolase activity, increase conversion of primary to secondary bile acids, reduce intestinal inflammation.	Indirectly modulates FXR and TGR5 by altering bile acid pool composition.	Animal models show improved lipid/glucose metabolism; small clinical trials in metabolic syndrome and liver diseases.	Preclinical data show reduced neuroinflammation and improved cognition via microbiota–BA–TGR5 signaling; may affect vagal activation, mood and stress responses. Potential regulation of appetite, satiety, and energy homeostasis via gut–brain axis; may influence vagal signaling.	Strain-specific effects; inter-individual variability; long-term efficacy unclear.	[178,283,284,285,286]
Prebiotics	Non-digestible fibers promote growth of beneficial microbes that metabolize bile acids, increase SCFA production, modulate gut pH, and reduce bile acids toxicity.	Indirect modulation of FXR/TGR5 through SCFA-mediated signaling.	Preclinical studies and limited clinical data show improved bile acid signaling and metabolic outcomes.	SCFA influence CNS via vagus nerve and enteroendocrine signaling, potentially affecting glucose homeostasis and appetite. SCFA-mediated enteroendocrine and vagal modulation may reduce CNS inflammation and enhance cognitive flexibility and appetite regulation.	Dose-dependent effects; specificity of prebiotic type; potential gastrointestinal side effects.	[287,288,289,290,291]
FXR Agonists (e.g., OCA, INT-747)	Activate FXR in liver and intestine; downregulate bile acid synthesis, upregulate transporters (BSEP, OSTα/β), reduce inflammation, regulate lipid/glucose metabolism. enhances insulin sensitivity.	Direct activation of FXR; suppresses CYP7A1; alters hepatic and intestinal bile acid pool.	Preclinical studies; early-phase clinical trials.	May influence CNS-mediated energy balance; indirectly improves glucose/lipid metabolism. Indirect CNS benefits by lowering systemic inflammation and bile acid-driven barrier dysfunction; FXR–FGF19 axis influences hypothalamic energy regulation and neuroinflammatory pathways.	Long-term safety unknown; limited receptor specificity; systemic effects possible.	[91,269,277,292,293,294]
FXR Antagonists	Block FXR signaling; increase hepatic bile acid synthesis and intestinal bile acid excretion, may enhance secondary bile acid production.	Decreased FXR-mediated repression of CYP7A1; modifies bile acids pool.	Mostly preclinical studies; limited human data.	Potential indirect effects on CNS and metabolism; research ongoing.	Risk of cholestasis; metabolic effects not fully characterized.	[295,296,297]
TGR5 Agonists (e.g., INT-777, betulinic acid, taurolithocholic acid)	Activate TGR5 on enteroendocrine cells; increase GLP-1 and PYY secretion, enhance energy expenditure, modulate bile acid signaling, reduce neuroinflammation, suppress hypothalamic neurons.	Direct TGR5 activation; enhances bile acid-mediated signaling in intestine and liver.	Preclinical studies; early clinical trials.	Neuroprotective and anti-inflammatory effects via microglial TGR5 activation; potential benefits in neurodegenerative disease models. Improves glucose homeostasis, influences appetite via CNS gut–brain axis, potential therapeutic target for Alzheimer’s disease.	Bioavailability and tissue specificity; potential cardiovascular effects.	[99,293,298,299,300]
TGR5 Antagonists	Inhibit TGR5 signaling; reduce GLP-1 secretion and energy expenditure, may attenuate bile acid-mediated metabolic effects.	Blocks TGR5-mediated GLP-1 release; modifies bile acid signaling.	Preclinical studies.	Potential therapeutic effect in polycystic liver disease; CNS effects unclear.	Limited human data; potential adverse impact on glucose metabolism; off-target effects possible.	[116,301]
Fecal Microbiota Transplantation	Transfers gut microbiota from healthy donors to recipients; restores microbial diversity, enhances bile acid metabolism, increases secondary bile acid production, modulates intestinal FXR/TGR5 signaling.	Alters bile acid pool composition; indirect modulation of FXR/TGR5 signaling.	Preclinical and clinical studies in metabolic syndrome, NAFLD, and IBD show improvement in bile acid profiles and metabolic parameters.	May improve CNS-regulated energy balance and metabolic outcomes via gut–brain axis.	Donor variability; safety concerns (infection risk); long-term efficacy uncertain.	[203,302,303,304]
TUDCA/UDCA (bile acid analogs)	Anti-oxidative and cytoprotective properties, anti-apoptotic activity, mitochondrial stabilization.	Improves bile acid homeostasis and reduces hydrophobic bile acid toxicity.	Clinical in ALS (TUDCA); preclinical in AD/PD models.	Robust neuroprotection in preclinical models: prevention of microglial activation, improved cognitive outcomes; ongoing clinical evaluation in ALS (TUDCA).	Variable CNS penetration; dose optimization needed.	[305,306,307]

The List of Abbreviations: ASBT—Apical Sodium-Dependent Bile Acid Transporter, BSEP—Bile Salt Export Pump, CNS—Central Nervous System, CYP7A1—Cholesterol 7α-Hydroxylase, DCA—Deoxycholic Acid, FXR—Farnesoid X Receptor, FGF19—Fibroblast Growth Factor 19, FGFR4—Fibroblast Growth Factor Receptor 4, GABA—Gamma-Aminobutyric Acid, GLP-1—Glucagon-Like Peptide-1, GR—Glucocorticoid Receptor, IBD—Inflammatory Bowel Disease, INT-747—Obeticholic Acid (6α-ethyl-chenodeoxycholic acid), JNK—c-Jun N-Terminal Kinase, KLB—β-Klotho, LCA—Lithocholic Acid, NAFLD—Non-Alcoholic Fatty Liver Disease, NPY—Neuropeptide Y, OCA—Obeticholic Acid, OATP—Organic Anion Transporting Polypeptide, OSTα/β—Organic Solute Transporter Alpha/Beta, PYY—Peptide YY, S1PR2—Sphingosine-1-Phosphate Receptor 2, SCFA—Short-Chain Fatty Acids, TGR5—Takeda G Protein-Coupled Receptor 5, TLR—Toll-Like Receptor, UDCA—Ursodeoxycholic Acid.

Bile acid-based therapies have advanced into clinical testing for neurodegenerative disease. Hydrophilic bile acids such as UDCA and TUDCA exhibit robust anti-apoptotic and neuroprotective effects in Alzheimer’s disease, Parkinson’s disease and amyotrophic lateral sclerosis (ALS) [12,308,309,310]. Phase II studies in ALS suggested that oral TUDCA, as add-on therapy, may slow functional decline, with acceptable safety profile [305,311]. A large multicenter phase III trial (TUDCA-ALS) is currently underway to rigorously test these preliminary signals using clinically meaningful endpoints (ALSFRS-R decline, survival) [305,309]. Beyond ALS, observational data suggest that UDCA administration use may be associated with reduced risk of age-related macular degeneration and other neurodegenerative outcomes, although causality and mechanism remain uncertain [312,313]. Thus, while preclinical data strongly support bile acid-based neuroprotective effects, current human evidence is still limited to small trials and observational cohorts, underscoring the need for large, well-powered randomized studies.

Rodent studies have implicated bile acids and TGR5/FXR signaling in hippocampal neurogenesis, synaptic plasticity and depression-like behavior [12]. Consistent with this, recent human studies have identified altered bile acid profiles and gut microbiota–bile acid interactions in depressive disorder [313]. A recent translational study showed that patients with depression exhibit gut microbiota dysbiosis with a shift in bile acid metabolism, which in turn promotes cognitive impairment via increased neuroinflammation and disrupted neurotrophic signaling [216,314,315]. Complementary imaging studies report associations between specific circulating bile acid species and functional connectivity within executive control networks and default mode networks in depressive disorder, suggesting that peripheral bile acid signaling may be linked to large-scale brain network organization in humans [316]. Although these findings resonate with mechanistic models derived from animals, they remain largely correlational, and interventional trials that directly manipulate bile acid signaling in psychiatric populations are still lacking.

Although mechanistic investigations in rodent models have elucidated many foundational pathways in bile acid-mediated gut–brain signaling, their translational applicability to humans is constrained by several important methodological issues. First, significant species differences in BA physiology exist. The composition of the bile acid pool differs markedly between humans and rodents, with important consequences for bile acid signaling, receptor activation and translational relevance. In humans, the primary bile acids are CA and CDCA, whereas in mice, the primary pool is dominated by muricholic acids (α-MCA, β-MCA, ω-MCA) in addition to CA. This divergence is functionally significant: muricholic acids act as FXR antagonists, while CDCA is one of the strongest FXR agonists, resulting in a fundamentally different basal tone of FXR signaling between species. Furthermore, humans predominantly conjugate bile acids with glycine, whereas rodents favor taurine conjugation, influencing solubility, microbial accessibility and receptor affinity. Secondary bile acid profiles are also species-specific. Humans generate high levels of DCA and LCA via microbial 7α-dehydroxylation, whereas rodents produce lower amounts of these hydrophobic species due to a distinct gut microbiota composition and differences in bile acid transport. As a result, TGR5 activation is generally lower in rodents, given the stronger affinity of TGR5 for LCA and DCA [317,318,319]. These interspecies differences mean that bile acid-mediated metabolic, immunological and neuroendocrine effects observed in rodent models cannot be directly extrapolated to humans without considering the underlying biochemical and microbial disparities that shape the bile acid pool. In addition, many interventions that modulate BA pools (such as dietary fiber, microbial modulation or pharmacologic receptor agonists) simultaneously affect peripheral metabolic, hepatic, microbial and immune systems. Consequently, separating direct central effects of bile acids from indirect peripheral mechanisms (e.g., improved insulin sensitivity, altered vagal afferent tone) remains challenging. Furthermore, human microbiome–bile acid studies are largely associative: interindividual variation in microbiome composition, changes in bile acid composition as a responses to dietary, microbiota or pharmacologic interventions contribute to the challenges in the clinical translation of preclinical data [320]. To strengthen the translation, future studies should adopt standardized human intervention designs, integrate CNS-specific endpoints (e.g., neuroimaging, cerebrospinal fluid bile acid profiles) and develop humanized animal models that recapitulate human bile acid composition. Addressing these limitations will enhance the therapeutic potential of targeting bile acid-driven gut–brain signaling axis for metabolic, neurodegenerative and psychiatric disorders.

## 10. Conclusions

Growing evidence demonstrates that bile acids function not only as digestive amphipathic molecules but also as powerful endocrine and neuroactive signals that shape metabolic and cognitive homeostasis through the gut–brain axis. By integrating microbial transformations of bile acids with neuronal, hormonal, and immune pathways, the gut microbiota establishes a biochemical communication system able to influence appetite regulation, glucose homeostasis, neuroinflammation, stress responses, and even higher-order brain processes. FXR- and TGR5-mediated signaling at intestinal, hepatic, and central levels represents the core of this regulatory network, while secondary bile acids and other microbial metabolites dynamically modulate its tone and direction. Dysregulation of these pathways, whether through changes in bile acid pool size, impaired receptor signaling, or microbial dysbiosis, contributes to the development of metabolic disorders, obesity, and neurodegenerative and neuropsychiatric diseases.

Therapeutic modulation of the gut microbiota–bile acid axis therefore offers a compelling opportunity for precision intervention. Current clinical data already support bile acid-based strategies in metabolic disease and neuroprotection, while novel agents targeting FXR, TGR5, and FGF19 signaling are emerging as promising tools for restoring disrupted gut–brain communication. However, important interspecies differences in bile acid composition and receptor biology highlight the need for human-focused mechanistic studies and carefully designed clinical trials.

By bridging microbiological, metabolic, and neuroendocrine perspectives, bile acid signaling emerges as a central integrator of host–microbe interactions with direct implications for systemic and brain health. Understanding and harnessing this axis opens a transformative path toward innovative diagnostics and therapies for complex disorders rooted in the gut–brain interface.

## Data Availability

No new data were created or analyzed in this study. Data sharing is not applicable to this article.
